# Dynamic Analysis of Integrated Signaling, Metabolic, and Regulatory Networks

**DOI:** 10.1371/journal.pcbi.1000086

**Published:** 2008-05-23

**Authors:** Jong Min Lee, Erwin P. Gianchandani, James A. Eddy, Jason A. Papin

**Affiliations:** Department of Biomedical Engineering, University of Virginia Health System, Charlottesville, Virginia, United States of America; King's College London, United Kingdom

## Abstract

Extracellular cues affect signaling, metabolic, and regulatory processes to elicit cellular responses. Although intracellular signaling, metabolic, and regulatory networks are highly integrated, previous analyses have largely focused on independent processes (e.g., metabolism) without considering the interplay that exists among them. However, there is evidence that many diseases arise from multifunctional components with roles throughout signaling, metabolic, and regulatory networks. Therefore, in this study, we propose a flux balance analysis (FBA)–based strategy, referred to as integrated dynamic FBA (idFBA), that dynamically simulates cellular phenotypes arising from integrated networks. The idFBA framework requires an integrated stoichiometric reconstruction of signaling, metabolic, and regulatory processes. It assumes quasi-steady-state conditions for “fast” reactions and incorporates “slow” reactions into the stoichiometric formalism in a time-delayed manner. To assess the efficacy of idFBA, we developed a prototypic integrated system comprising signaling, metabolic, and regulatory processes with network features characteristic of actual systems and incorporating kinetic parameters based on typical time scales observed in literature. idFBA was applied to the prototypic system, which was evaluated for different environments and gene regulatory rules. In addition, we applied the idFBA framework in a similar manner to a representative module of the single-cell eukaryotic organism *Saccharomyces cerevisiae*. Ultimately, idFBA facilitated quantitative, dynamic analysis of systemic effects of extracellular cues on cellular phenotypes and generated comparable time-course predictions when contrasted with an equivalent kinetic model. Since idFBA solves a linear programming problem and does not require an exhaustive list of detailed kinetic parameters, it may be efficiently scaled to integrated intracellular systems that incorporate signaling, metabolic, and regulatory processes at the genome scale, such as the *S. cerevisiae* system presented here.

## Author Summary

Cellular systems comprise many diverse components and component interactions spanning signal transduction, transcriptional regulation, and metabolism. Although signaling, metabolic, and regulatory activities are often investigated independently of one another, there is growing evidence that considerable interplay occurs among them, and that the malfunctioning of this interplay is associated with disease. The computational analysis of integrated networks has been challenging because of the varying time scales involved as well as the sheer magnitude of such systems (e.g., the numbers of rate constants involved). To this end, we developed a novel computational framework called integrated dynamic flux balance analysis (idFBA) that generates quantitative, dynamic predictions of species concentrations spanning signaling, regulatory, and metabolic processes. idFBA extends an existing approach called flux balance analysis (FBA) in that it couples “fast” and “slow” reactions, thereby facilitating the study of whole-cell phenotypes and not just sub-cellular network properties. We applied this framework to a prototypic integrated system derived from literature as well as a representative integrated yeast module (the high-osmolarity glycerol [HOG] pathway) and generated time-course predictions that matched with available experimental data. By extending this framework to larger-scale systems, phenotypic profiles of whole-cell systems could be attained expeditiously.

## Introduction

Intracellular biochemical networks are comprised of signaling, metabolic, and regulatory processes. (Note that here we use “regulation” to refer specifically to transcriptional regulatory and protein synthesis networks, and “signaling” to describe intracellular reactions that drive responses to the extracellular environment.) Until recently, computational analyses focused independently on signaling, metabolic, and regulatory networks. However, high-throughput experimental data coupled with computational systems analysis techniques have elucidated multifunctional components involved in fundamental disease processes [1]–[4]. For example, signaling cascades are triggered by the presence of extracellular stimuli and often result in activation of transcription factors. These transcription factors function in regulatory networks, regulating the transcription of associated genes and the synthesis of various proteins used in signal transduction and metabolism. Cellular metabolism is responsible for the production of energy in the form of adenosine triphosphate (ATP) and the synthesis of amino acids among other biomass precursors, all of which are used elsewhere in the cell. Consequently, a key challenge in the post-genomic era is to consider the interconnectedness of biochemical networks and how extracellular cues affect highly integrated intracellular processes to elicit cellular responses such as growth or differentiation.

Dynamic [5],[6] and structural analyses [7] have been employed to quantitatively analyze large-scale biochemical network modules. Typically, in dynamic analyses, a set of ordinary differential equations (ODEs) describing the mass (balance) of each species in the system is constructed. Despite its generality, the application of this type of mechanistic model at a genome-scale is largely considered impractical because it necessitates the consideration of many pathways for which detailed reactions and their kinetic parameters are not yet known. Structural analyses like flux balance analysis (FBA) can calculate phenotypic properties of a biological network like a steady-state flux (i.e., reaction rate) distribution without detailed kinetic information. FBA requires a physiologically relevant objective function (e.g., in the case of metabolism, maximizing the growth rate or maximizing ATP production), mass-balance constraints (i.e., the stoichiometry of the reactions), and constraints on reaction directions and thermodynamics. Since the physicochemical constraints are readily defined (e.g., from the annotated genome sequence and measured enzymatic capacities), FBA has been used effectively to study large-scale biochemical networks, particularly metabolic networks [8]. However, in general, the steady-state assumption of FBA prevents it from generating dynamic concentration profiles of intracellular species.

An additional challenge to the modeling of *integrated* systems is that time scales of intracellular biochemical networks generally span multiple orders of magnitude. Signaling and metabolic reactions typically occur rapidly. For example, kinase/phosphatase reactions, protein conformational changes, and most metabolic reactions occur on the order of fractions of a second to seconds [9]. By contrast, receptor internalization [10] and regulatory events [11],[12], as well as end-stage phenotypic properties such as cellular growth or differentiation [13] can take several minutes to hours. These multiple time-scales pose computational challenges for the quantitative analysis of integrated systems. For instance, kinetic model-based strategies suffer from a scarcity of values for kinetic parameters as well as poor accuracy of known kinetic parameters [14]. In addition, models of integrated systems are inherently “stiff,” i.e., they must include “fast” and “slow” reaction dynamics simultaneously [15], and they are consequently difficult to simulate and extremely sensitive to modeling errors [16]. Indeed, it is challenging to apply FBA to models of integrated systems because of the steady-state assumption intrinsic to FBA and the “fast” and “slow” reaction dynamics that coexist intracellularly. Due to these complexities, previous models and analyses have focused primarily on network modules rather than integrated systems. These include kinetic, stoichiometric, and causality analyses of modular signaling systems [17]–[20], metabolism [21]–[25], and regulation [26],[27].

Some preliminary dynamic analyses of integrated systems have been completed. Integrated analyses of regulatory and metabolic networks revealed novel mechanisms in *Saccharomyces cerevisiae* and *Escherichia coli* [28]–[30]. Metabolic reactions were represented stoichiometrically, and regulatory reactions were captured by representing gene regulatory rules using a Boolean formalism. FBA was implemented assuming quasi-steady-state conditions, i.e., the typical time constant of metabolic transients was relatively faster than the simulation time step for temporal integration of phenotypic variables (e.g., biomass as a measure of cellular growth). Recently, a kinetic model accounting for signal transduction, metabolism, and regulation was constructed to describe the response of *S. cerevisiae* to osmotic shock [31]. This model connected specific outputs of one network (e.g., signaling) with the inputs of another network (e.g., metabolism) in a “sequential” fashion. The complete set of interactions among the biochemical networks, such as feedback and feed-forward of proteins expressed as a function of the regulatory network to signaling and metabolism, was not considered. Additionally, it required an exhaustive list of kinetic parameters (e.g., rate constants) for the reactions, and time-courses of individual modules (or collections of reactions) were evaluated separately. As reconstructions of large-scale signaling and metabolic networks are emerging, there is a growing need for the development of a framework to study these networks from an integrated perspective [9].

The purpose of this study was to develop a FBA-based computational framework, termed integrated dynamic Flux Balance Analysis (idFBA), for the quantitative, dynamic analysis of cellular behaviors arising from signaling, metabolic, and regulatory networks at the genome-scale. The idFBA framework requires an integrated stoichiometric reconstruction of signaling, metabolic, and regulatory processes. It assumes quasi-steady-state conditions for “fast” reactions and incorporates “slow” reactions in a time-delayed manner. To assess the efficacy of idFBA, we developed a prototypic integrated system with topological features characteristic of those observed in existing signaling, metabolic, and regulatory network reconstructions as well as kinetic parameters reported in literature. Additionally, we applied in a similar manner the idFBA framework to a representative module in *S. cerevisiae* as a validation of our approach. idFBA allowed for quantitative, dynamic analysis of systemic effects of extracellular cues on phenotypes of these systems and generated comparable time-course predictions when contrasted with kinetic models. Ultimately, we demonstrate how idFBA enables genome-scale quantitative, dynamic analysis of integrated systems.

## Methods

The idFBA framework facilitates the dynamic analysis of cellular phenotypes on the genome scale arising from extracellular cues. The systems evaluated as part of this study, including an integrated prototype spanning signaling, metabolism, and regulation, and a representative module from yeast are described here. The implementation details of the framework are also delineated.

### Biological Systems Evaluated: Prototypic Integrated System

**Figure 1:**
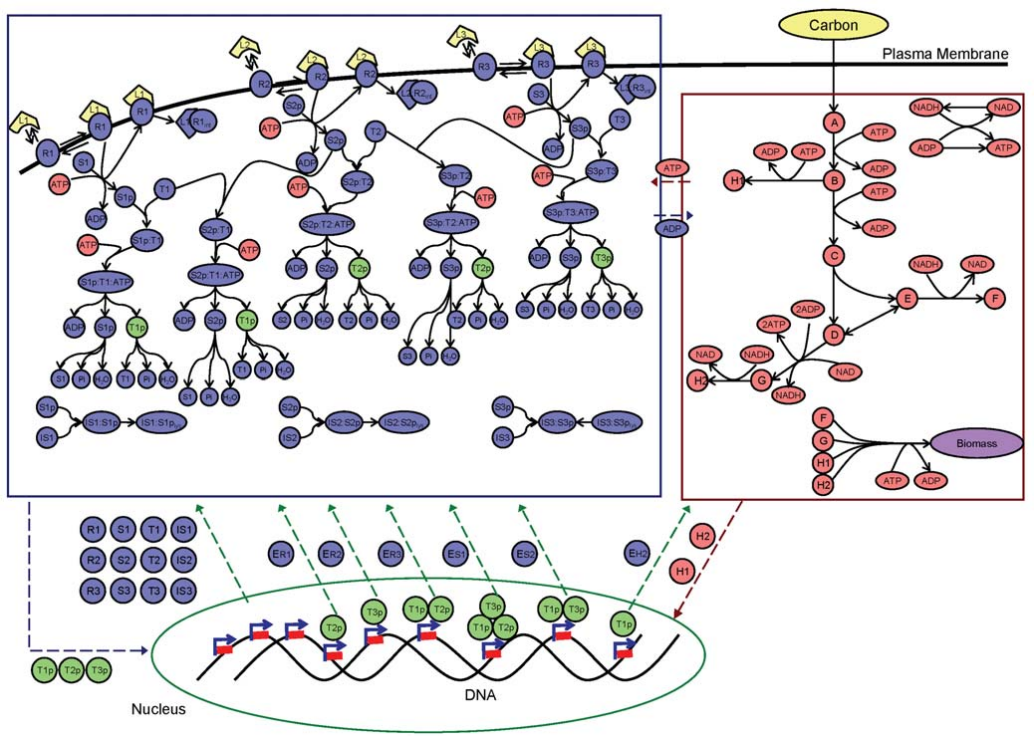
The prototypic integrated system. The prototypic integrated system, comprised of integrated signaling, metabolism, and regulation, is illustrated. Solid boundary lines indicate the three functional network modules: signal transduction (upper left), metabolism (upper right), and transcriptional regulation (bottom). Dashed lines between the modules represent interactions spanning multiple modules, arising from compounds that simultaneously participate in reactions of different functional modules. The components and reactions within these networks are based on published network reconstructions of actual biological systems. Primary roles of network components are shaded by color: blue for signaling, red for metabolism, and green for regulation. Detailed reactions are presented in Text S1.

**Figure 2:**
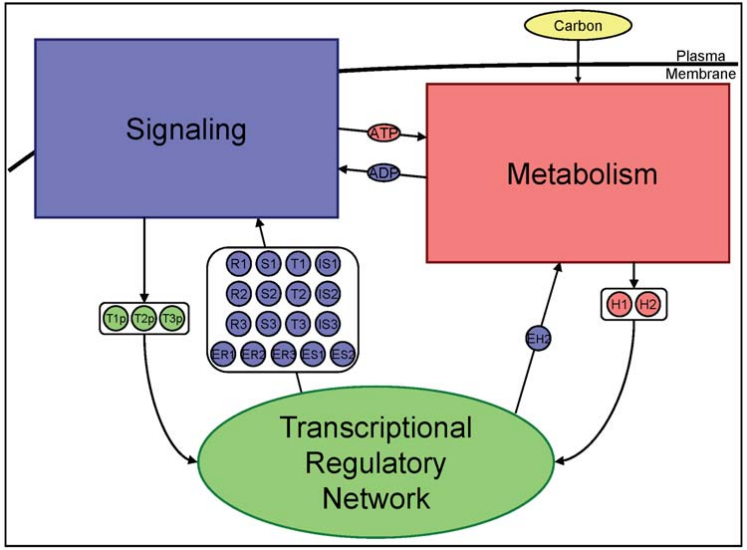
The degree of interconnectivity across signaling, metabolism, and regulation in the prototypic integrated system. Network components with overlapping functions across the three functional network modules of signal transduction, metabolism, and transcriptional regulation are illustrated. Primary roles of the network components are shaded by color: blue for signaling, red for metabolism, and green for regulation. (See Table 3 for a listing of the input/output relationships within the prototype.)

In order to assess the efficacy of idFBA, a prototypic integrated system was constructed with characteristics typical of those observed in published reconstructions of signaling, metabolic, and regulatory networks (see Figures 1 and 2). Specifically, we generated representative reactions with stoichiometric relationships and estimated their associated rate constants from literature. Here we briefly describe the reactions that are considered in each network and their typical time scales. Detailed information on these reactions and associated kinetic parameters is provided in Text S1.

#### Signal transduction

Signal transduction pathways govern a cell's response to extracellular stimuli, including, e.g., how a cell adapts its transcriptional regulatory program in response to specific environmental cues. The prototypic signaling network is comprised of a set of reactions that attempts to mimic what is typical of biological signaling pathways such as phosphorelay and kinase cascade modules. As shown in Figure 1 (top left), ligands (L_1_, L_2_, and L_3_) bind to receptors (R_1_, R_2_, and R_3_) to form ligand-receptor complexes (L_1_R_1_, L_2_R_2_, and L_3_R_3_). These complexes are subsequently either internalized or involved in phosphorylation events. Phosphorylation of signaling components takes place through a series of reactions involving ATP and other activated components. Any one signaling component can also activate multiple other signaling components; this activity represents the type of multi-functionality (e.g., crosstalk) that is often found in biological systems [32]. Ultimately, activated transcription factors (T_1_p, T_2_p, and T_3_p), that are representative of phosphorylated proteins are the downstream effector molecules that result from the signaling pathways.

The model of signal transduction consists of a total of 45 reactions. As previously described, the rate constants for these reactions are based on values observed for similar signaling reactions in literature [10],[18],[31]. Most of the reactions in the prototypic signaling network are “fast” relative to transcriptional regulation; steady-state concentrations are achieved on the order of seconds. However, there are some “slow” reactions that take on the order of several minutes to hours to reach steady state. These include the internalization of ligand-receptor complexes and inhibition and hydrolysis of activated components (see Text S1). The typical order of magnitude of the concentrations of signaling components in this prototypic integrated system is micro-molar (µM) [18],[31].

#### Metabolism

Metabolic pathways produce energy, amino acids, and other precursors required for the growth and maintenance of a cell. The metabolic reactions in the prototypic system comprise pathways representative of glycolysis and amino acid synthesis (see Figure 1, top right). The model contains 13 reactions, and the associated kinetic parameters were adapted from previous work [21],[22],[31]. The biosynthetic requirements for cellular growth (i.e., biomass production) were defined based on the prototypic metabolic reactions defined in [29] (see Equation 1), where H_1_ and H_2_ are representative of amino acids and F and G are representative of metabolites.
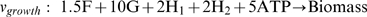
(1)The maximum carbon utilization rate, *S_u_* ^max^, was set to 10.5 mmol/(g(dry weight)•h) as in [24].

Most of the metabolic reactions in the model are “fast” and achieve steady states in several seconds. The growth of biomass is on the order of hours. The typical order of magnitude of metabolite concentrations is milli-molar (mM) [22].

#### Regulation

Transcriptional regulatory networks control the transcription state of a genome. In general, they describe the connections between environmental cues and transcriptional responses [1]. Inputs to regulatory networks are environmental cues, including the presence and absence of extracellular metabolites, reaction fluxes, and specific conditions such as pH values. The internal reactions, often not known in chemical detail, are represented by regulatory rules that describe the activation or inhibition of gene transcription in response to these environmental cues. The outputs are the synthesized protein products that result through a combination of the signaling inputs acting upon the regulatory rules as well as consequent transcription and translation.

These networks have been mathematically described using a Boolean formalism, in which the state of a gene is represented as either transcribed or not transcribed in response to regulatory signals [1]. This formalism employs Boolean operators such as AND, OR, and NOT to describe the dependence of gene transcription upon extracellular metabolites and transcription factors as in [29]. Recently, a formalism that represents such regulatory rules in matrix form was developed, allowing for the systemic characterization of the properties of a transcriptional regulatory network and facilitating the computation of the transcriptional state of the genome under any given set of environmental conditions [1]. Furthermore, this “quasi-stoichiometric” matrix formalism enables regulatory networks to be represented alongside stoichiometric representations of signaling and metabolic networks: if a gene is repressed, fluxes of reactions involving the corresponding protein product are constrained to zero.

Studies on the dynamic behavior of regulation have involved constructing mass-balanced models of messenger RNA (mRNA) transcripts, ribosomes, and proteasomes in order to quantitatively predict protein synthesis [26],[31]. However, these approaches require estimation of rate constants that are difficult to measure experimentally. Furthermore, these descriptions of regulation are not complete because they do not account for the amino acids produced from metabolism and required for protein synthesis. In order to effectively couple regulation with other functional cellular modules, a more complete representation of the dynamic behavior of protein synthesis that facilitates balancing of input/output relationships across network modules is required.

The goal of the idFBA approach presented here, therefore, is to quantitatively account for the production and use of proteins throughout the cell. The transcriptional regulatory network is comprised of transcription factors that associate with specific genes, leading to the activation or inactivation of gene transcription. Activated genes yield proteins that participate in various intracellular signaling, metabolic, and regulatory reactions. Additionally, we considered amino acid requirements for protein synthesis: typically 30–80 moles of amino acids were required for every mole of protein, as shown in Table 1 [33]. Kinetics of protein synthesis were modeled as a second-order reaction between two amino acids H_1_ and H_2_ (*k_poly_*[H_1_][H_2_]), and the kinetic parameter *k_poly_* was estimated by considering a typical time constant for protein production based on [28]. The concentrations of mRNA transcripts, ribosomes, and proteasomes were assumed to be constant, and their effects on protein synthesis were captured by *k_poly_* (for the purposes of our model, we assumed that only amino acids could contribute mass to protein production).

**Table 1:** Amino acid requirements (H_1_ and H_2_) for synthesis of protein (*a_i_*H_1_+*b_i_*H_2_ → Protein*_i_*).

Protein*_i_*	*a_i_*	*b_i_*	Protein*_i_*	*a_i_*	*b_i_*
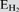	10	20	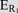	15	15
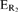	10	25	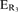	20	10
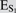	13	18	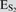	18	13
R_1_	20	40	S_1_	30	30
T_1_	25	35	IS_1_	35	35
R_2_	25	45	S_2_	30	40
T_2_	25	55	IS_2_	35	45
R_3_	15	55	S_3_	15	25
T_3_	20	20	IS_3_	10	30

The variables 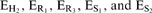 represent the enzymes for the reactions synthesizing H_2_, R_1_, R_3_, S_1_, and S_2_, respectively, within the metabolic network. The other proteins can be found in Figure 1 and participate in signaling functions.

The prototypic transcriptional regulatory network presented here is comprised of 18 genes (see Figure 1, bottom). Three transcription factors are inputs to the system, and 18 protein products with functions in metabolism and signaling are outputs of the network. Of the 18 genes, six are regulated by the presence or absence of the transcription factors. The remaining genes are defined to be constitutively active. The transcriptional regulatory rules for the six regulated genes are described using a Boolean formalism, as in [1] and [29]. For example, the regulatory rule in Equation 2 implies that Gene ER_3_ is expressed only if both T_1_p and T_2_p are present.
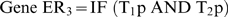
(2)The complete set of transcriptional regulatory rules for the prototypic integrated system is defined in Table 2. For simplicity, the amino acid requirements for protein synthesis are only considered for the proteins indicated in Table 1; however, similar requirements could be implemented as desired.

**Table 2:** Regulatory rules for the transcriptional regulatory network of the prototypic integrated system.

Expression of a gene	Regulation
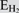	If (T_1_p)
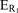	If (T_2_p)
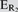	If (T_3_p)
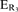	If ((T_1_p) AND (T_2_p))
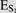	If ((T_1_p) AND (T_2_p) AND (T_3_p))
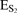	If ((T_1_p) AND (T_3_p))

A total of six genes are regulated by three transcription factors in the prototypic regulatory system. The Boolean regulatory rules for these six genes over these three transcription factors are presented.

#### Interactions and mixed-time scale model

As previously described, a cellular phenotype ultimately arises from complex interactions of network components across signaling, metabolism, and regulation. The prototypic integrated system described above was designed to exhibit the interconnectedness seen in actual cellular systems, as illustrated by the input/output relationships between the three functional modules of signaling, metabolism, and regulation (see Figure 2 as well as Table 3). Furthermore, kinetic rate constants for the prototypic system were collected from representative reactions in literature (as reported in Text S1), and consequently the prototypic system exhibits dynamics across multiple time scales as seen *in vivo*.

**Table 3:** Input/output relationships in the prototypic integrated system.

	Input	Output	Time scale
**Signal transduction**	LigandsEnergyProteins	Activated transcription factors	Fast & Slow
**Metabolism**	CarbonProteins	EnergyAmino acidsBiomassProteins	Fast & Slow
**Transcriptional regulation**	Activated transcription factorsAmino acids		Slow

The degree of interconnectivity across signaling, metabolism, and regulation in the prototypic integrated system is summarized (see Figure 2 for a graphical depiction). Inputs to and outputs from each of the three biochemical networks contained within the prototypic integrated system are noted. Ultimately, the prototype considers feedback and feed-forward across signaling, metabolism, and regulation.

### Biological Systems Evaluated: Representative Module in *S. cerevisiae*

**Figure 3:**
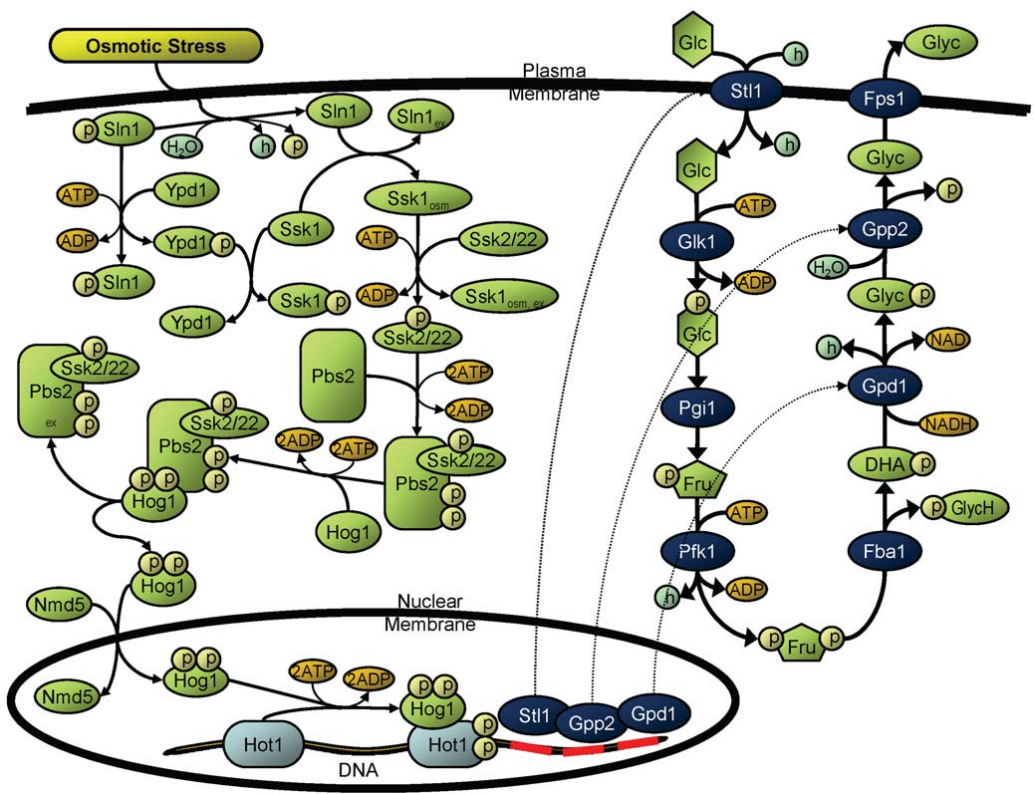
A representative integrated module in *S. cerevisiae*. A representative integrated module in *S. cerevisiae*, the high osmolarity glycerol (HOG) pathway, is illustrated. Signaling reactions appear in the upper left of the figure, metabolic reactions in the upper right, and regulatory reactions in the bottom. The components and reactions within these networks are based on published literature on the *S. cerevisiae* HOG pathway. Detailed components and reactions are presented in Text S2.

To assess the applicability of idFBA to actual biological systems, a representative integrated module in *S. cerevisiae*, the prototypic single-cell eukaryote, was investigated. This module was comprised of key aspects of yeast osmoregulation, i.e., the active processes with which yeast cells monitor and adjust pressure and control their shape, turgor, and water content in response to extracellular conditions [34]. The signaling, metabolic, and regulatory activities included in this module are illustrated in Figure 3.

Specifically, we reconstructed a portion of the high-osmolarity glycerol response (HOG) pathway, one of four major mitogen-activated protein (MAP) kinase cascades in *S. cerevisiae*, from existing literature. The HOG MAP kinase pathway plays a pivotal role in the adaptation of *S. cerevisiae* to conditions of high external osmolarity [35]. For example, yeast cells deficient in this pathway cannot proliferate on media containing high levels of osmotically active molecules [34]–[36]. Extensive genetic analysis has previously been performed, leading to experimental identification of many activating and inhibiting components of the HOG signaling pathway [37]. In general, yeast cells use the HOG pathway to accumulate glycerol under hyperosmotic conditions, to balance the osmotic pressure with the extracellular environment. Osmotic stress signals are communicated via the HOG signaling pathway, leading to the activation of Hot1 and other transcription factors. These transcription factors subsequently promote the expression of glycolytic enzymes, such as Stl1, Gpd1, and Gpp2, thereby catalyzing metabolic reactions leading to increased glycerol production.

As this model serves an illustrative purpose here, the HOG pathway was restricted to the key set of reactions necessary for its phenotypic function. Specifically, 26 reactions spanning 48 components were assimilated in stoichiometric matrix form, including 16 reactions across 33 components in signaling; a single transcription factor activating three regulated genes; and seven reactions across 12 components in metabolism. Inputs of this module included osmotic shock (signaling) and glucose (metabolism), and outputs included glycerol (metabolism). Key reactions connecting the underlying signaling, metabolic, and regulatory processes were the translocation of the kinase Hog1 into the nucleus for the activation of transcription factor Hot1 (signal transduction and metabolism), and the synthesis of metabolic enzymes Stl1, Gpd1, and Gpp2 for reactions involved in the conversion of glucose to glycerol (transcriptional regulation and metabolism). Other reactions in the HOG pathway as previously experimentally characterized (e.g., inhibition of Hog1 by phosphatases Ptp2, Ptp3, and Ptc1, thereby allowing the cell to keep the HOG pathway in check and maintain osmotic balance) were excluded from the reconstruction used here for simplicity.

As with the prototypic system, the representative integrated yeast module was implemented using the idFBA framework as well as a kinetic model similar to the one in [31], and the two approaches were contrasted for validation purposes. Rate constants describing the kinetics of the system were culled from available experimental data, notably [31]. For complete details of the reconstructed yeast HOG pathway, including listings of reactions, rate constants, and kinetic equations, see Text S2.

### Flux Balance Analysis

One modeling technique for evaluating cellular phenotypes is called flux balance analysis (FBA). FBA is a constraints-based approach that attempts to derive a phenotype in the form of a steady-state flux distribution for the reactions in a given biological system. FBA is based on the principle that all expressed phenotypes of a given biological system must satisfy basic constraints that are imposed on the functions of all cells [8],[38],[39]. These constraints are physico-chemical (i.e., physical laws like conservation of mass and energy); topological (i.e., spatial restrictions on metabolites within cellular compartments); and environmental (i.e., nutrient availability, pH, and temperature, all of which vary over time and space) [8],[20],[38]. Because FBA yields fluxes rather than concentrations, limited kinetic information is required for its implementation.

FBA requires a stoichiometric reconstruction of the biochemical network of interest. An annotated genome cataloging which reactions specific enzymes catalyze is the basis for a detailed description of a network's components and interactions [40]. This biochemical network reconstruction can be represented in matrix form, **S**, where **S** is of size *m* components×*n* reactions and is comprised of stoichiometric coefficients that capture the underlying reactions of the biochemical network.

After the network is reconstructed, fluxes are calculated by deriving a dynamic mass balance for all the components within the system [7],[38]. Specifically, at steady state, the change in the amount of a component *C* over time *t* across all reactions within the system must be zero. Consequently, mass balance is defined in terms of the flux through each reaction and the stoichiometry of that reaction, and a set of coupled ordinary differential equations relating the roles of reactions with components may be written in the form of Equation 3.
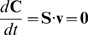
(3)Here, **S** denotes the *m*×*n* matrix of stoichiometric coefficients and **v** denotes the vector of *n* reaction fluxes, with each element (row) of the *n*-row vector **v** corresponding to the flux in the associated reaction (column) in **S**. The vector **C** is a *m*-row vector defining the concentrations of the *m* components within the system. This mass balance represents the principal constraint in FBA and defines a feasible solution space for the set of fluxes. Additional constraints such as thermodynamics can be incorporated into FBA as well, further narrowing the possible distribution of fluxes [24],[41].

Equation 3 generally leads to an under-determined system because the number of components tends to be far fewer than the number of reactions. Even with additional constraints, FBA usually requires performing an optimization with linear programming (LP) to identify a particular flux distribution. In other words, FBA involves optimizing the set of fluxes such that the flux through a particular cellular reaction is maximized (or minimized). A cellular objective represents what a given biological system has optimized toward through evolutionary pressures [42]. It is defined as a linear equation (Equation 4), where **c** is the vector that defines the coefficients, or weights, for each of the fluxes in **v** [41].
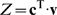
(4)This general representation of *Z*, wherein the elements of **c** can be easily manipulated, enables the formulation of many diverse objectives. Common choices for cellular objective functions in models of metabolic networks include biomass production [24],[43], energy [44], and byproduct production [45].

Ultimately, FBA attempts to solve the LP problem in Equation 5 to find a physiologically-relevant cellular phenotype in the form of a flux distribution **v** that optimizes *Z* while lying in the bounded solution space defined by a set of physio-chemical, topological, and environmental constraints.
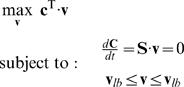
(5)Note that **v** *_lb_* and **v** *_ub_* are the lower and upper bounds on the reaction fluxes, respectively. For example, thermodynamic constraints or reaction directionalities can be incorporated by setting a given *v_lb_* = 0.

Though the steady-state assumption of FBA precludes the calculation of dynamic concentrations of the network components, dynamic profiles of cellular phenotypes (e.g., cellular growth or differentiation) have been successfully predicted with a quasi-steady-state assumption [24],[25],[29]. This assumption involves discretizing the time domain into intervals, and (1) solving the LP problem contained within FBA at the beginning of each interval, and (2) based on the resultant flux data, solving a system of ODEs for concentrations over time within each interval.

Applications of FBA to dynamic simulations have focused on metabolic networks because time constants of metabolic transients are typically very rapid when contrasted with time constants characterizing whole-cell phenotypic changes. Exceptions include the incorporation of gene regulatory events, which are much slower than metabolic reactions, into FBA for time-course simulation of metabolic reactions [28],[29]. In these cases, the regulatory constraints were described as Boolean operators and imposed in a time-delayed manner. However, these examples are limited to metabolic and regulatory processes and do not consider changes in the mass balance (e.g., protein synthesis) arising from the interactions between metabolic and regulatory processes and signaling systems. Consequently, quantitative, dynamic analyses of integrated cellular systems have not been explored in detail, limiting the characterization of whole-cell function.

### idFBA: An FBA-Based Approach for the Dynamic Simulation of Integrated Systems

As previously described, the stoichiometric reconstruction enforces explicit, chemically-consistent accounting of the components and reactions underlying a biochemical network, and facilitates the systematic analysis of fundamental network properties with FBA and associated analysis techniques [32]. The stoichiometric reconstruction and FBA are particularly applicable to large-scale networks, for which a lack of kinetic data (e.g., rate constants) makes kinetic-based approaches impractical. Indeed, stoichiometric reconstruction and FBA have been applied successfully to large-scale metabolic and signaling networks, elucidating characteristics of these networks [8],[9].

Therefore, integrating signaling reactions with metabolic and regulatory reactions using FBA can facilitate the dynamic analysis of cellular phenotypes arising from environmental cues and provide a complete snapshot of cellular sysems. However, as previously described, applying FBA directly to integrated networks is challenging. First, unlike metabolic systems in which objectives for the FBA formulation are often experimentally characterized (e.g., the production of biomass), objectives of signaling and regulatory systems are not well-defined. Second, integrated networks are comprised of reactions with mixed time scales (e.g., signaling reactions are generally much faster than regulatory reactions), and FBA has previously been applied only to fast reactions for which steady-state assumptions hold.

Here we describe the idFBA framework, including how we address these challenges. We use the prototypic integrated system as the basis for this discussion.

#### FBA-based representation of signaling networks

**Figure 4:**
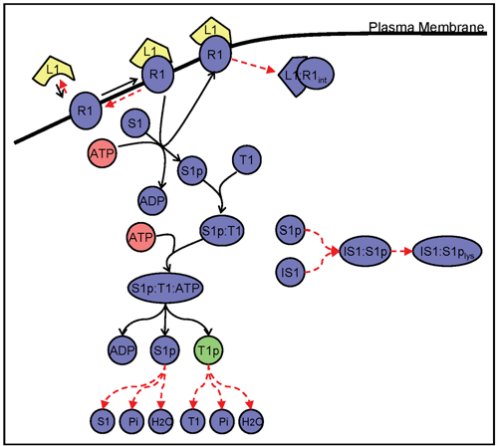
A representative signaling pathway in the prototypic integrated system. One of the pathways of the prototypic signaling network is illustrated. Here, solid black lines represent reactions that have non-zero fluxes, while dotted red lines represent reactions that have zero fluxes when the production of the activated transcription factor T_1_p is maximized as the pathway objective.

As previously described, we represent signaling networks using a stoichiometric formalism, and we calculate a flux distribution with FBA (see Equation 5). Transcription factors activate transcriptional regulatory programs in response to extracellular cues. Consequently, one choice for the objective of a signaling network is maximizing the activation of transcription factors. However, as illustrated for the prototypic signaling network depicted in Figure 4, this objective by itself fails to generalize to a feasible flux distribution. Instead, maximizing the activation of the transcription factor T_1_p consistently yields zero fluxes for the key pathway reactions denoted by dashed lines, including receptor internalization, pathway inhibition, and transcription factor degradation.

To address this challenge, we model the objective of a signaling network by introducing a binary parameter, represented as the matrix **I**(*R_i_*, *t*). **I**(*R_i_*, *t*) indicates whether reaction *R_i_* is to be included in the system at time *t*, given an underlying network objective. It is constructed on the basis of a set of rules and other parameters (e.g., the time that is required for receptor internalization or protein synthesis and degradation, etc.) that a user specifies as consistent with data about the given system. For a given reaction *R_i_* at time *t*, **I**(*R_i_*, *t*) is multiplied by the upper bound of the associated reaction flux *v_i_*. If a particular reaction is included in the network at time *t* based on the user-defined rules and parameters (i.e., it has a non-zero flux at that time), the binary parameters **I**(*R_i_*, *t*) for the reactions sharing components with the included reaction are set to zero at that time point and/or at future time points, depending on the specified time delays, indicating that they are not included in the network. Multiplying these zero-values by the upper bounds of the associated reaction fluxes nets a new upper bound of zero. As a consequence, fluxes through the reactions sharing components with an included reaction are set to zero at specific times in order to drive all the flux through the included reaction. In this way, the hypothesized network objective is maximized, all the while ensuring that flux is driven through all “active” reactions. For example, in Figure 4, including the receptor internalization reaction (L_1_R_1_ → L_1_R_1,int_) implies that the binary variables for the reactions (L_1_R_1_+S_1_ → L_1_R_1_ · S_1_) and (L_1_R_1_ → L_1_+R_1_) are set to zero. In this manner, a feasible flux distribution for a signaling network is obtained by maximizing for the activation of transcription factors. Importantly, the binary parameter **I**(*R_i_*, *t*) can take into account time delays associated with “slow” reactions, as described below.

#### Incorporation of slow reactions into FBA

In addition, to characterize mixed time-scale phenomena using FBA, we implement idFBA by assuming quasi-steady-state conditions for “fast” reactions and incorporating “slow” reactions into the stoichiometric matrix in a time-delayed manner as in [29]. In other words, we approximate continuous phenomena occurring over long time as instantaneous events at particular time points. Two parameters are used to implement this approach: time-delay (τ*_delay_*), indicating after what time a “slow” reaction is considered an “active” steady-state constraint in the stoichiometric matrix; and reaction duration (τ*_duration_*), indicating how long the “slow” reaction remains as the effective constraint once it is activated. In the prototypic integrated system, “slow” reactions include protein degradation, pathway inhibition, and receptor internalization in the signaling network; the uptake of a carbon source and production of biomass in the metabolic network; and the synthesis of proteins in the transcriptional regulatory network.

#### Dynamic simulation of integrated systems

The optimized flux distribution that results from FBA is used to predict the time-course of phenotypic variables. The time-scale separation between “slow” and “fast” reactions is determined by the discretization of the time domain. Specifically, a reaction that reaches steady state or that produces a product at a specified threshold concentration within a single time step is considered “fast.” “Slow” reactions are those that take longer than the unit time interval to attain steady state.

**Figure 5:**
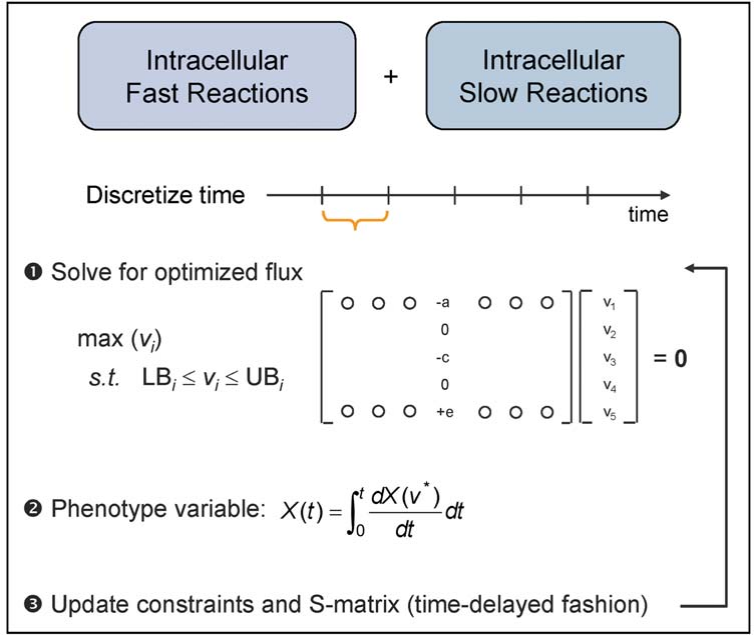
The idFBA framework. The key steps in the idFBA framework are summarized. Specifically, the time window is discretized into small steps, Δ*t*. FBA is used to calculate a flux distribution through the network (1), phenotypic variables such as cell density at timepoint *t_current_* are evaluated by integrating the resultant flux values over the time step Δ*t* (2), and the fluxes and phenotypic variables are used to update constraints for the next time step (3). Part of step 3 involves updating an incidence matrix (I) denoting which reactions participate during which time steps of the simulation.

Ultimately, the implementation of the idFBA framework can be described as a seven-step process (see Figure 5):

1. Discretize the time window into small steps, Δ*t*. For example, in the case of the prototypic integrated system, the time step was specified as 0.1 h as described in “Results” below.
2. Initialize a *R_s_*×*t_N_* incidence matrix (**I**) denoting which reactions participate during which time steps (Equation 6). Here *R_s_* represents the number of reactions within the system and *t_N_* the number of time intervals (see Equation 6).
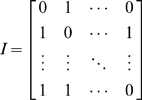
(6)Each row of **I**(*R_i_*, *t*) denotes a reaction *R_i_*, and each column denotes a time step Δ*t*. The coefficients of **I**, at the intersection of reactions and time steps, are binary parameters indicating whether a given reaction participates during a given time step. A “0” denotes that a given reaction does not participate in the system at the specified time step, whereas a “1” denotes that the reaction does participate in the system at that time step. Although **I** is difficult to generate for an actual biological system given the limitations of available experimental technologies, it facilitates a best-guess of the system dynamics based on available literature. For example, for any given system, **I** can be derived from experimental data and assumptions inputted into the idFBA framework.
3. For each reaction in the system *R_i_*, multiply the corresponding coefficient **I**(*R_i_*, *t*) by the flux bounds of the reaction. By specifying **I**(*R_i_*, *t*) = 0 for excluded reactions, the fluxes of these reactions are set to zero when a “slow” reaction is included. For example, consider the signaling network shown in Figure 4. If the internalization reaction [L_1_R_1_] → [L_1_R_1,int_] is included and “active” and the objective of the network is maximizing the production of the activated transcription factor T_1_p, fluxes of the excluded reactions [L_1_R_1_]+[S_1_] → [L_1_R_1_ · S_1_] and [L_1_R_1_] → [L_1_]+[R_1_] are set to zero at the associated time steps.
4. Solve Equation 5 for the optimized flux vector, **v**, with the updated constraints, for the start of the current time step, *t_current_*.
5. Given the optimized flux vector for *t_current_*, integrate the phenotype variable, *X_p_*, over the time step Δ*t* (Equation 7).
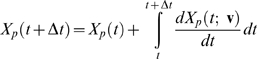
(7)Here we consider two phenotype variables, namely cell density (*X*) and substrate concentration (*S_c_*). These terms are given by Equations 8 and 9, where μ is a specific growth rate, and *S_u_* is the uptake rate for the carbon source.
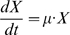
(8)

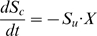
(9)
6. Update **I** based on **v** at the current time step *t_current_* given the time-delay and reaction duration parameters (τ*_delay_ and* τ*_duration_*, respectively) (Equation 10).
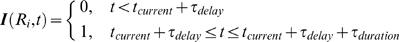
(10)Specifically, as previously described, the dynamic parameters τ*_delay_* and τ*_duration_* approximate the progression of “slow” reactions as steady-state constraints in the **idFBA** framework. The parameter τ*_delay_* describes when a particular “slow” reaction appears as a steady-state constraint in the stoichiometric matrix and instantaneously becomes an “active” reaction. The parameter τ*_duration_* indicates how long the “slow” reaction is kept as a constraint in the stoichiometric matrix and maintained active. For example, in the transcriptional regulatory network, if the reaction flux of a transcription factor exceeds a specified threshold, the transcription of its target gene is incorporated into the matrix after a defined time (τ*_delay_*) (here τ*_delay_* mimics the delay for protein synthesis, including transcription and translation), and the protein is assumed to remain in the system until it degrades (a period of time captured by τ*_duration_*).
7. Repeat steps 3 through 6. The optimized flux vector, **v**, at the current time step *t_current_* imposes new constraints on the internal fluxes of the next time step. These constraints include ligand binding rates, carbon uptake rate, and protein production rates.

As described above, implementing the idFBA framework in this manner dynamically simulates cellular phenotypes arising from integrated biochemical networks. We describe the results for a prototypic integrated system as well as a representative yeast module below.

Technical implementation details. idFBA was implemented on the prototypic and yeast integrated systems in MATLAB v. 7.5 (part of the MathWorks R2007b release package).

### Kinetic Modeling

To validate the results of the idFBA framework, we developed kinetic models of the prototypic integrated system and the representative integrated yeast module. As previously stated, kinetic models describe the temporal changes of compound concentrations due to production, degradation, modification, or transport [46]. In other words, the rate of change of the concentration *C_i_* of the *i^th^* compound within a system may be described as in Equation 11 below [46]. Here *S_ij_* is the stoichiometric coefficient, *v_j_* is the rate of the *j^th^* reaction, and *n* is the total number of reactions in the network. Reactions that produce or consume the *i^th^* compound have non-zero stoichiometric coefficients and are therefore included in the *i^th^* differential equation.
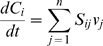
(11)The reaction rates for the network, **v**, are functions of component concentrations, such as the concentrations of enzymes (e.g., kinases and phosphatases within a signaling network), as well as parameters including kinetic constants. These rates are described by different types of kinetic laws. For example, Michaelis-Menten expressions can be used to model enzyme kinetics [47],[48].

Our ODE models of the prototypic integrated system and representative integrated yeast module were constructed from the underlying reaction network, with rate constants (i.e., kinetic parameters) obtained from literature. The systems of ODEs were continuously solved over the time window of interest (equivalent to that of the corresponding idFBA implementations). Details of these models, including the kinetic equations, kinetic constants, and ordinary differential equations, are presented in Text S1 and S2.

It is important to note that idFBA and kinetic modeling constitute two independent approaches. The idFBA framework involves performing an optimization, over multiple discretized time steps, to approximate the dynamics of a system with time-delay information from strictly stoichiometric constraints. By contrast, a kinetic model requires all of the kinetic parameters and, by continuously solving a set of ordinary differential equations, yields a more detailed portrait of the system dynamics. We attempt to illustrate here how the idFBA framework, with significantly fewer parameters, approximates the system dynamics observed through much more detailed ODE models.

Technical implementation details. The kinetic models of the integrated prototypic system and representative integrated yeast module were implemented using the ode23tb ODE solver in MATLAB v. 7.5 (part of the MathWorks R2007b release package). The ode23tb solver is an implementation of an implicit Runge-Kutta formula, comprised of a trapezoidal rule followed by a backward differentiation formula of order two. The solver compromises efficiency for crude tolerances [49].

## Results

Using the prototypic integrated system shown in Figure 1, predictions of the dynamic characteristics of phenotypic variables (i.e., cellular growth and substrate consumption) were made for different conditions. We demonstrate how ligand availability and changes in regulatory rules affect the phenotype behavior. We also assess the suitability of our approach by comparing the idFBA results with a corresponding kinetic model of the same system. Furthermore, we summarize results for a representative integrated yeast module.

### Prototypic Integrated System

#### Implementation details

The specific implementation of the idFBA framework on the prototypic integrated system is detailed below.

1. The sample time, Δ*t*, was set to 0.1 h as in [24]. This time step was chosen to account for typical reaction kinetics across different cellular processes.
2. The maximum carbon uptake rate, 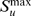, was set to 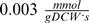 as is observed in *E. coli* [24].
3. Constraints on the uptake of substrates from the extracellular environment were required in order to identify an optimal flux distribution through the metabolic network. These constraints are detailed in Text S1. Similarly, ligand binding rates were necessary to calculate a flux distribution (facilitating the evaluation of “active” and “inactive” species) through the signaling network. We assumed rate constants for the ligand binding reactions (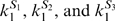) as well as ligand and receptor concentrations that were similar to published parameters [10],[18],[31]. Thus, ligand binding rates were evaluated (e.g., 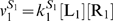).
4. Temporal parameters (namely τ*_delay_* and τ*_duration_*) were specified to account for “slow” reactions. The “slow” reactions were allowed to participate in the reaction network after a delay τ*_delay_* and with duration τ*_duration_*. The following rules were applied at time *t*:
5.
  - The binary variable **I**(*R_i_*, *t*) corresponding to the reaction *R_i_* describing the synthesis of a particular protein was set to 1 (indicating the reaction was “active”) if the flux of the activated transcription factor exceeded a specified threshold (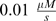 as in [29]). Because FBA does not directly compute intracellular concentrations, we used specific flux values as thresholds here. There exists precedent in the literature for this approach [29],[50]. For example, if the stability of a given transcription factor is low, the flux corresponding to the activation of that transcription factor would have to be very high in order for transcriptional effects to occur. Furthermore, experimental data recently demonstrated that, for any gene, the amount of protein synthesized correlates well with the transcription rate *up to* about one-third of the maximal transcription rate for that gene [51],[52]. Beyond that point, much greater noise in protein production was observed as a function of gene transcription rate. Consequently, an experimentally-measured gene transcription rate (or a rate-based threshold) may serve as an appropriate quantitative predictor of whether a reaction catalyzed by the corresponding protein product should be allowed to occur at a given time step within the idFBA framework. Similarly, specific metabolic reaction fluxes have been experimentally measured under multiple conditions and used to characterize flux thresholds that must be attained in order for cell growth (and other phenotypes) to occur (see [53] for an example of how this was recently completed in *Trypanosoma brucei*). All these data support the use of flux thresholds in idFBA, and these types of measurements would serve as inputs to the idFBA framework in future implementations.
  - If the flux of a phosphorylated component was not zero (i.e., if the component was considered to be in an “active state”), elements of **I** for inhibition and degradation of the component were set to 1 (indicating these reactions were “active”) after specified time delays (τ*_delay_*) and with durations (τ*_duration_*) of one sample time. This particular τ*_duration_* was chosen since the steady-state constraints of these reactions impose complete depletion of available reactants within the current sample time *t_current_*. Similarly, elements of **I** for the internalization of ligand-receptor complexes were set to 0 (indicating these reactions were “inactive”) after a time delay accounting for the time it takes for the complexes to become internalized.
6. The objective functions of the resultant FBA formulations included maximizing the production of: (1) activated transcription factors in the signaling network; (2) the set of metabolites that produce biomass in the metabolic network; and (3) the amino acids, in relative ratios, that are necessary for the synthesis of proteins by the transcriptional regulatory network. The fluxes for the activation of transcription factors are 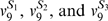 (see Text S1). Biomass production (Equation 12, top) and protein synthesis (Equation 12, bottom) were approximated as the single reaction in Equation 13, where *a_i_* and *b_i_* are specified in Table 1.
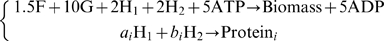
(12)

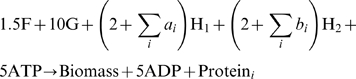
(13)Hence the objective function for the prototypic integrated system corresponded to Equation 14.
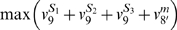
(14)

The optimized fluxes for the production of activated transcription factors (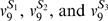) were indicative of the activation of associated genes and 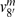 was used for calculating cell growth and carbon uptake according to Equations (8) and (9) with 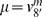. The flux 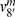 at a single time step further constrained the protein synthesis reaction fluxes during the subsequent time step.

As we describe subsequently (see “Discussion”), a method called Biological Objective Solution Search (BOSS) was recently developed for the inference of an objective function for a biological system from its underlying network stoichiometry as well as experimentally-measured flux distribution [54]. Therefore, aside from approximating the objective function in the manner described above, utilizing BOSS to identify objectives for the signaling, regulatory, and metabolic networks would facilitate the identification of an *in silico* flux distribution for the integrated system, a key step in the idFBA framework.

#### Evaluating effects of environmental cues

To evaluate the utility of the idFBA framework, the phenotypic characteristics of the prototypic integrated system were evaluated under a variety of different conditions. First, the dependence of cellular growth on different combinations of input ligands L_1_, L_2_, and L_3_ was assessed. Table 4 shows the parameters τ*_delay_* and τ*_duration_* representing the typical temporal characteristics of “slow” reactions [10],[18],[28].

**Table 4:** “Slow” reactions in the prototypic integrated system.

Description	Reactions	Excluded reactions	τ_del_ (min)	τ_dur_ (min)
Degradation of phosphorylated compounds	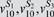	—	—	40
Degradation of transcription factors	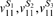	—	—	40
Production and degradation of additional proteins	*v^R^*	—	40	40
Internalization of ligand receptor complexes	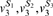	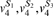 , 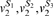	40	6
Inhibitory reactions	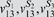	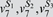 , 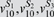	40	6

The superscript *S* denotes signaling reactions. For example, *S*_1_ represents the set of reactions associated with the ligand L_1_ (See Figure 1). The fluxes of excluded reactions in a given optimization instance are set to zero when the corresponding reactions participate in the network.

**Figure 6:**
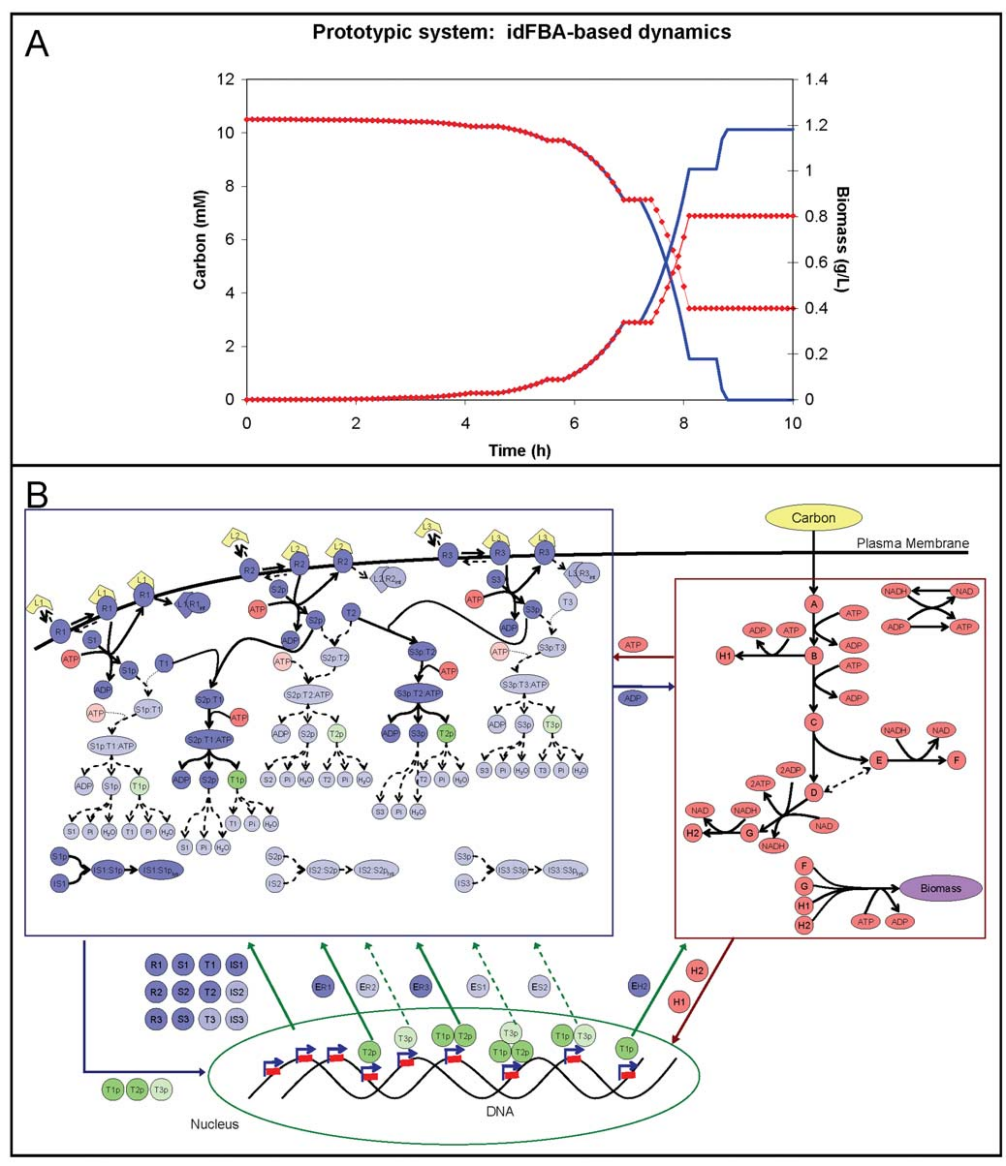
Dynamic profiles of the prototypic integrated system. In (A), the concentration of carbon and amount of biomass within the cellular system over a simulation time of 10 h is illustrated. The decreasing lines represent the concentration of carbon (as indicated by the left *y*-axis), which is being consumed, in the system. The increasing lines represent the amount of biomass, which is being synthesized, in the system (as indicated by the right *y*-axis). The blue solid lines correspond to the case in which the ligand concentrations are set to [L_1_] = [L_2_] = [L_3_] = 2 µM during the simulation. By contrast, the red dotted lines correspond to the case in which no ligand is present during the time 6.0 h≤*t*≤6.5 h. In (B), the fluxes through the system at *t* = 6.7 h for this second scenario in which none of the ligands is present during the time 6.0 h<*t*<6.5 h are presented. Here the solid lines represent non-zero flux values and the dotted lines represent zero flux values. Additionally, components that do not participate in this scenario are shaded lightly.

We first simulated the case in which the concentration of all three ligands was 2.0 µM. The results are shown in Figure 6A (blue solid lines). The carbon source was completely depleted by *t* = 8.7 h from an initial concentration (or “dose”) of 10.5 mM. The production of the amino acid H_2_ was catalyzed by the enzyme 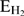 with an initial delay of τ*_delay_* = 40 min, and consequently, cellular growth was sluggish during this initial period. Two periods of no growth (i.e., at approximately *t* = 7 h and *t* = 8.25 h) corresponded to times when enzymes that catalyze metabolic reactions and protein synthesis were unavailable. For example, the first phase of no growth at *t* = 7 h was due to the degradation (and therefore inactivity) of transcription factors regulating key factors involved in biomass production, and the second at *t* = 8.25 h was caused by the degradation of phosphorylated proteins (e.g., S_1_p) that activate transcription factors leading to protein synthesis. Although these types of on/off descriptions are not precise, they serve as useful approximations of the phenotypic behavior over an entire time course.

We subsequently simulated the case in which the ligands were temporarily unavailable for cellular uptake during the evaluated time-course (see Figure 6(A), red dotted lines). Specifically, no ligand was available for cellular uptake at 6.0 h≤*t*≤6.5 h. Consequently, a no-growth period was observed at about 7 h. All transcription factors generated before *t* = 6 h were degraded by this time, preventing the amino acid H_2_ from being synthesized for a period of 0.5 h (i.e., until the ligand supply was restored). The cell also stopped growing at about *t* = 8.2 h. The transcription factor T_3_p, which activates the synthesis of enzyme 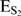 in the prototypic integrated system, was not produced, leading to a lack of synthesis of the protein S_1_. Additionally, the simultaneous absence of all ligands led to the inactivation of S_1_p (S_1_) and the consequent lack of phosphorylation of T_3_ as illustrated in Figure 6(B). Note that, by contrast, both T_1_p and T_2_p have additional activation pathways (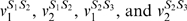), and the genes whose expression is dependent upon these transcription factors were still transcribed.

We also tested the effects of individual ligands or groups of ligands on cell growth by evaluating the phenotypic characteristics for the input cases described in Equation 15. For example, we considered the effect of ligand L_1_ by itself by restricting the availability of ligands L_2_ and L_3_ beyond t = 2 h. Similarly, we further assessed the effect of ligand L_1_ by restricting its availability while maintaining the concentrations of ligands L_2_ and L_3_ beyond t = 2 h.
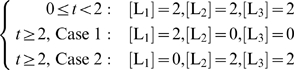
(15)


**Figure 7:**
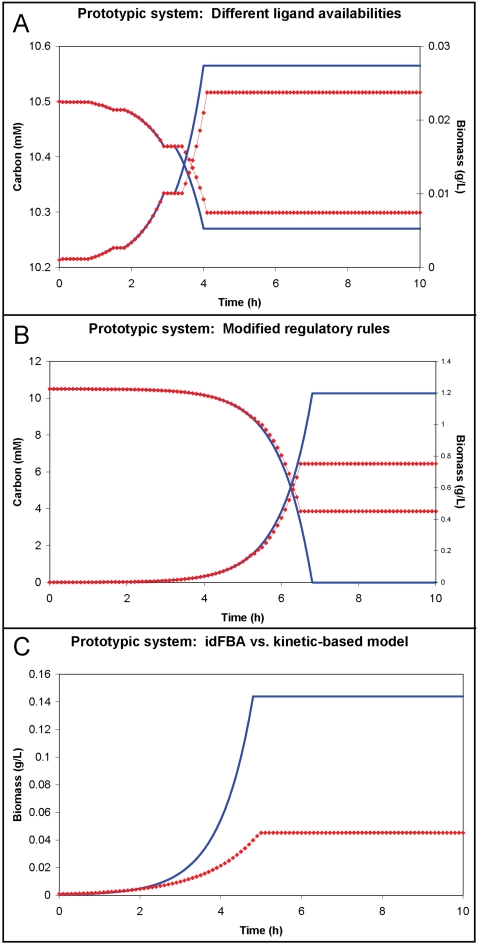
Dynamic profiles of the prototypic integrated system under different conditions, and a comparison to the kinetic-based model. The concentration of carbon and amount of biomass within the cellular system over a simulation time of 10 h is illustrated. The decreasing lines represent the concentration of carbon (as indicated by the left *y*-axis), which is being consumed, in the system. The increasing lines represent the amount of biomass, which is being synthesized, in the system (as indicated by the right *y*-axis). (A) depicts the idFBA results when the system is subjected to two different sets of ligand availabilities. The blue solid lines correspond to case 1, i.e., [L_2_] = [L_3_] = 0 µM for *t*≥2 h, and the red dotted lines correspond to case 2, i.e., [L_1_] = 0 µM for *t*≥2 h. In (B), the behavior of the prototypic integrated system under the modified regulatory rules shown in Table 5 is presented. The blue solid lines correspond to ligand concentrations of [L_1_] = [L_2_] = 2 µM and [L_3_] = 0 µM, and the red dotted lines correspond to ligand concentrations of [L_1_] = [L_2_] = [L_3_] = 0 µM for 5.0 h<*t*<7.0 h. In (C), the dynamics of cellular growth generated by the idFBA framework are contrasted by those specified by an equivalent detailed kinetic-based model over a simulation time of 10 h. Here the blue solid line corresponds to the amount of synthesized biomass specified by the idFBA framework, and the red dotted line corresponds to the amount of synthesized biomass specified by the detailed kinetic-based model.

As illustrated in Figure 1, the transcription factor T_1_p activates the gene corresponding to the enzyme 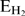, which catalyzes the production of H_2_, an essential amino acid for protein synthesis and, consequently, cellular growth. T_1_p is produced through a series of reactions in the prototypic integrated system. Specifically, the ligand L_1_ initiates a series of reactions leading to the production of T_1_ and its eventual phosphorylation (T_1_p). The inactive T_1_ can also be phosphorylated via a series of reactions initiated by ligand L_2_ if T_1_ is present in the system. Figure 7(A) illustrates that, when L_1_ is present, approximately 15 percent more biomass is produced (case 1, blue solid line). This result makes sense since L_1_ initiates greater production of T_1_ than L_2_, which is involved in the synthesis of both T_1_ and T_2_. Eventually, both cases failed to produce biomass or uptake carbon after *t* = 4 h: in case 1, the absence of T_2_p and T_3_p meant that key receptors for the uptake of carbon were not expressed; and in case 2 (red dotted lines), the absence of T_1_p meant that the enzyme synthesizing the key amino acid H_2_ was no longer expressed. These types of dynamic characteristics of complex ligand availabilities are as expected yet are difficult to analyze without considering all the interactions in an integrated, quantitative manner such as the one implemented by idFBA.

#### Effects of regulatory rule modifications

Changes to the regulatory program were evaluated as well. A new set of Boolean regulatory rules was implemented, as shown in Table 5. Specifically, an additional rule specifying that both T_1_p and T_2_p together, and not T_1_p or T_2_p individually, are required for the production of the protein F was incorporated into the regulatory network. Again, two scenarios were simulated. In the first one, no ligand was available to the cell at 5≤*t*≤7 h. In the second one, the transcription factor T_3_p was not synthesized at 5≤*t*≤7 h. With these exceptions, the concentration of all ligands was maintained at 2 µM. Figure 7B illustrates that, when both T_1_p and T_2_p are available, the carbon supply is exhausted and maximum biomass is attained at *t* = 6.7 h, down from *t* = 8.7 h under the original regulatory program (blue solid line). By contrast, when no ligand is present during the two hours, the cellular machinery is unable to consume all of the carbon supply and instead the amount of biomass it synthesizes is reduced by over 33% (Figure 7B, red dotted line).

**Table 5:** Modified regulatory rules.

Expression of a gene	Regulation
IS_1_	If (T_1_p)
IS_2_	If (T_2_p)
IS_3_	If (T_3_p)
E_F_	If NOT ((T_1_p) AND (T_2_p))
E_INT_	If ((T_1_p) AND (T_2_p) AND (T_3_p))

To evaluate how the idFBA framework performs under different sets of regulatory rules, a new set of Boolean rules for the transcriptional regulatory network was defined. These rules are summarized here.

#### Comparison to a kinetic-based model

The idFBA framework, as applied to the prototypic integrated system, was compared to a kinetic model that represented the reactions as ordinary differential equations. For the kinetic model, representative kinetic parameters were obtained from literature, as detailed in Text S1. As previously described, these two approaches are completely independent: the idFBA framework requires only stoichiometric constraints and approximates the dynamics of the system with time-delay information, whereas the kinetic model requires all of the kinetic parameters and yields a more detailed portrait of the system dynamics. For both implementations, we assumed an initial ligand concentration, 2.0 µM, for all three ligands. We note that the following dynamic parameters for slow reactions were identified from the kinetic model and implemented as τ*_delay_* and τ*_duration_* in idFBA: the degradation of transcription factors, 5 h, the delay in protein synthesis, 40 min, the degradation of proteins, 4 h, internalization, 5 h, and inhibition, 5 h. One striking result is shown in Figure 7C. The growth times calculated by both approaches are comparable (computed as 4.9 h for idFBA (blue solid line) and 5.1 h for the kinetic model (red dotted line)), with a difference of just two time steps over a 51-time-step simulation. The discrepancy in the amount of biomass synthesized is a consequence of the kinetic-based model itself. Unlike in idFBA which accounts for transcriptional regulation, all of the reactions in the metabolic network of the kinetic model are constitutively active. As a result, resources such as amino acids are used in other pathways, e.g., for the synthesis of surplus proteins, and consequently the amount of biomass produced is less than the value estimated by idFBA which simply maximizes for biomass production. One way to overcome this challenge is to further discretize the time domain in the idFBA implementation. In other words, as the level of discretization is increased (i.e., the length of each time step is decreased), the predictive precision of the idFBA framework improves, and vice-versa. At the same time, this increase in predictive precision must be balanced by an increase in computational complexity due to the additional calculations that are necessitated. Nevertheless, as illustrated with the prototypic system (and the representative integrated yeast module below), idFBA effectively approximates the dynamics of a system using purely the underlying network stoichiometry, efficaciously offering novel hypotheses that can serve as the basis for further experimental and computational study.

#### Robustness to parameter values

**Figure 8:**
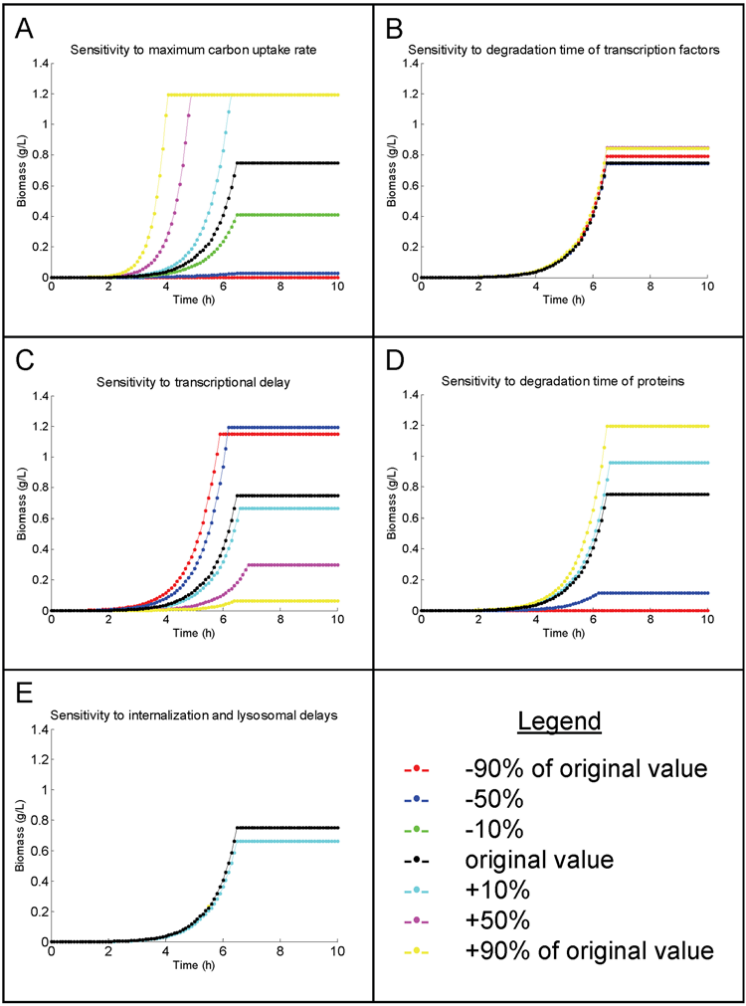
Robustness of parameter values in the prototypic integrated system. We evaluated the sensitivity of the idFBA-based implementation of the prototypic integrated system to specific parameter values. (A–E) illustrate the sensitivity of the amount of biomass synthesized and amount of carbon consumed to the maximum carbon uptake rate, the degradation time of the transcription factors, the time delay due to transcription and translation, the degradation time of the proteins, and the time delay due to receptor-ligand internalization as well as lysosomal effects, respectively. Note that each graph includes plots for the original parameter value as well as 10%, 50%, and 90% variation in both directions (up and down), as described in the legend.

To further assess the practicality of the idFBA framework at a large scale, we systematically evaluated how robust the framework was with respect to each of several parameters for the prototypic integrated system. Specifically, we considered the maximum carbon uptake rate, 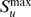, as well as the different time delays imposed on the system. In the case of the prototypic system, these delays included the degradation time of the transcription factors, the time delay due to transcription and translation, the degradation time of the proteins, and the time delay due to receptor-ligand internalization as well as lysosomal activity. For each of these five parameters, we varied the initial value used for the simulations described above by 10%, 50%, and 90% in each direction (up and down) and observed the resultant phenotypic variables, i.e., the concentrations of carbon and biomass, over time. The results of this robustness analysis for biomass synthesis are illustrated in Figure 8 (carbon consumption data not shown). The system was robust to changes in the degradation time of transcription factors and the delays associated with receptor-ligand internalization and lysosomal processing (Figure 8B and 8D, respectively). The change in the total amount of biomass synthesized was less than 12% for up to 90% change (up or down) in the values of these parameters. By contrast, changes to the maximum carbon uptake rate, the transcriptional delay, and the protein degradation time altered the time course of biomass synthesis more noticeably (Figure 8A, 8C, and 8D, respectively), suggesting that increasing accuracy in these parameters corresponds to increasing confidence in the idFBA-based results. Similar observations were noted with the amount of carbon consumed under these varying conditions (results not shown). Interestingly, these results matched well with our expectations. For example, when the length of the transcriptional delay is decreased significantly (by 50% or 90% of the original value), the time it takes to attain maximal biomass falls as well (Figure 8C, blue and red lines, respectively, versus black line). This finding further strengthens our confidence in the idFBA-based implementation of the prototypic integrated system. Note that we performed our robustness analysis on the prototypic integrated system with modified regulatory rules described above (see Table 5 for the modified regulatory rules and Figure 7B for the results of this system when the original parameter values were specified).

In general, robustness analyses facilitate an understanding of which parameters are most critical in determining overall system behavior. Parameters for which the system is particularly sensitive should be accurately inputted into the idFBA framework. Experimental protocols for measuring parameter values are improving. For example, substrate uptake rate can be determined by monitoring the depletion of the substrate source in filtered media samples over time using enzymatic assays or liquid chromatography. [55]. Likewise, the temporal details of a metabolic transcription program were recently evaluated [56]. Furthermore, on a still larger scale, several methods have been proposed recently for parameter estimation in biochemical pathways [57]–[62].

Additionally, robustness analyses can systematically establish *a priori* which model variables are reliably predicted by the idFBA framework for a given implementation. For example, those variables whose values change the least in response to perturbations in all of the model parameters are robust to the idFBA-based implementation. In the case of the prototypic integrated system, both biomass and carbon source do not fluctuate significantly in response to smaller variations in the parameter values, whereas the profiles of other species' concentrations are altered more significantly (results not shown).

### Representative Yeast Module

#### Implementation details

The implementation of the idFBA framework on the *S. cerevisiae* HOG pathway was similar to that of the prototypic integrated system described above. Key aspects of this implementation are detailed below.

1. The sample time, Δ*t*, was set to 0.1 h as in [24] to account for typical reaction kinetics across different cellular processes.
2. The maximum carbon uptake rate, 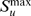, was set to 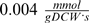 as previously observed in *S. cerevisiae* [63].
3. To obtain optimal flux distributions in metabolism and signaling (facilitating the evaluation of “active” and “inactive” species), rates for the uptake of carbon and signal transduction of osmotic stress were chosen based on known experimental values [31]. The associated constraints are detailed in Text S2.
4. Temporal parameters (namely τ*_delay_* and τ*_duration_*) were specified to account for “slow” reactions as in the idFBA implementation of the prototype. Briefly, “slow” reactions were allowed to participate in the reaction network after a delay τ*_delay_* and with duration τ*_duration_*. At any given time *t*, the binary variable **I**(*R_i_*, *t*) governed whether a particular protein was present based on the flux of activated transcription factor Hot1 [29].
5. The objective functions of the FBA formulations for the yeast system included maximizing the production of: (1) the activated transcription factor in the signaling network; (2) the metabolite that produces biomass (for the purposes of this reconstruction and analysis, glycerol) in the metabolic network; and (3) the amino acids (assumed to be derived from glycerol in relative ratios) that are necessary for the synthesis of the three proteins Stl1, Gpd2, and Gpp1 by the transcriptional regulatory network. The flux for the activation of the single transcription factor Hot1 is 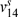, and the flux for the synthesis of biomass (glycerol) is 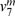. Consequently, Equation 16 constitutes the single composite objective function for the representative integrated yeast module.
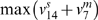
(16)

The optimized flux for the activation of transcription factor Hot1 (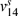) was indicative of the activation of the target genes Stl1, Gpd1, and Gpp2, and 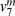 was used for calculating cell growth and determining glycerol accumulation in response to osmotic stress according to Equations 8 and 9 with 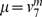. The flux 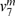 at a single time step further constrained the protein synthesis reaction fluxes during the subsequent time step. As mentioned above and further described below, a recently-developed framework called BOSS may be used in future analyses to more precisely identify objectives for the signaling, regulatory, and metabolic networks [54].

#### Observations and comparison to a kinetic-based model

**Figure 9:**
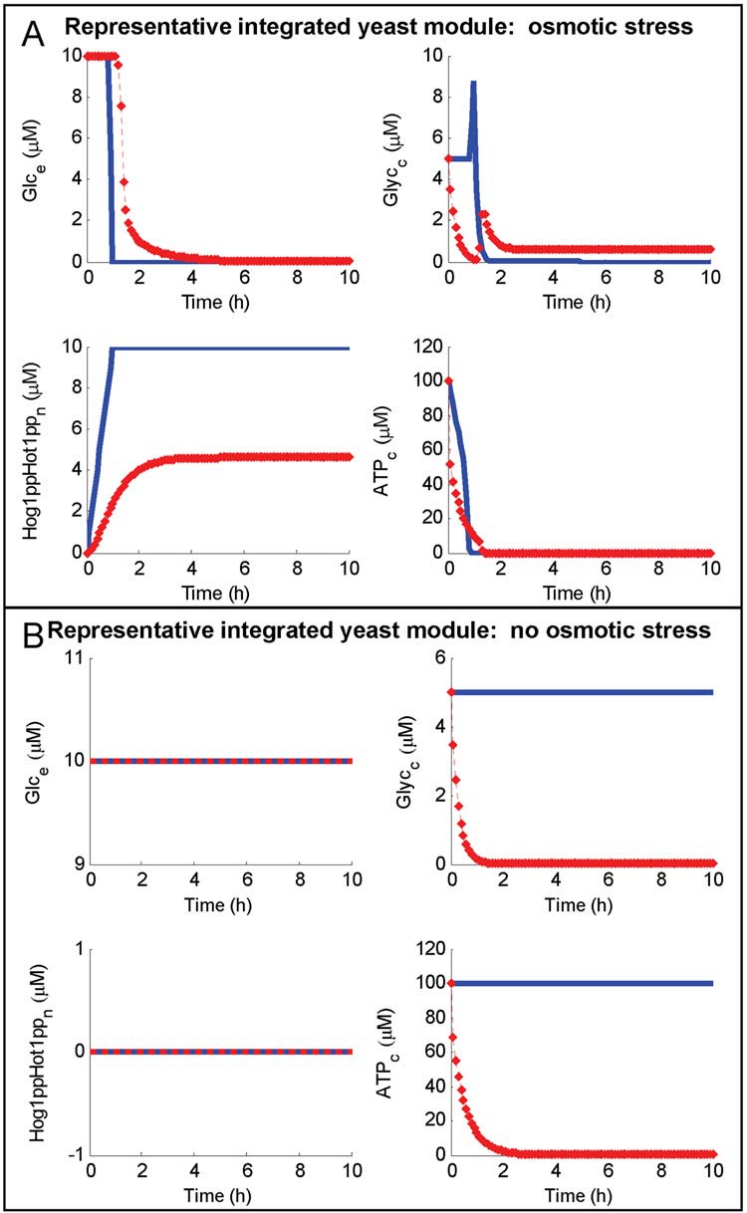
Dynamics of key species in the representative integrated yeast module as calculated by the idFBA framework and contrasted with a detailed kinetic-based model. The idFBA and kinetic-based model dynamics of glucose, glycerol, the activated transcription factor Hot1, and cytosolic ATP are contrasted over a simulation time of 10 h. Here, the blue solid line corresponds to the idFBA framework and the red dashed line corresponds to the detailed kinetic-based model. (A) and (B) correspond to situations of osmotic stress and no osmotic stress, respectively.

To evaluate the representative integrated yeast module using the idFBA framework, the phenotypic characteristics of the system were investigated under two conditions, i.e., the presence and absence of osmotic stress due to the cell-environment interaction. The idFBA results are shown in Figure 9 (blue solid lines). In the case of osmotic stress, the *in silico* yeast cell responded by synthesizing metabolic proteins essential for the conversion of glucose to glycerol. Additional glycerol accumulated within the cell between 1.5 and 2 h post-stimulation by osmotic stress (see Figure 9A), an observation consistent with published experimental findings [37]. By contrast, when osmotic stress was not present, the Hog1 signaling pathway did not activate Hot1, metabolic genes Stl1, Gpd2, and Gpp1 were not expressed, and no additional glycerol was synthesized (see Figure 9B).

Additionally, as illustrated in Figure 9, the dynamics of the yeast system described by the idFBA framework (blue solid lines) were in reasonable accord with those described by the equivalent kinetic-based model (red dashed lines). For example, in Figure 9A, both simulations suggested an initial delay in protein synthesis, likely due to transcriptional delays, followed by a rapid increase in glycerol concentration at between 1.5 and 2 h post-stimulation by osmotic stress. As stated above for prototypic system, differences in specific values of the different network components is a consequence of the kinetic-based model itself in which, unlike idFBA, reactions are constitutively active as implemented herein. For instance, in Figure 9(B), the kinetic-based model suggests that an initial intracellular concentration of glycerol (5 µM) is exported out of the cell via the glycerol exchange reaction (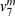); however, idFBA does not illustrate this result because all metabolic fluxes are constrained to zero at each time step since no glucose is taken up by the cell. As described previously in the context of the prototypic integrated system, increasing the level of discretization of the time window (i.e., decreasing the length of each time step) for the representative yeast integrated module yields improves the accuracy of the concentration values generated by the idFBA framework (results not shown).

Ultimately, this validation of the idFBA-based implementation of the representative integrated yeast module implies that it may be used to further probe the *S. cerevisiae* HOG pathway. Additional work may include (1) a sensitivity analysis of the model parameters and (2) an evaluation of the effects of network perturbations, such as single- or double-gene (reaction) knockouts. This type of analysis at the whole-cell level bridges the gap in knowledge of how systemic phenotypes arise in response to extracellular conditions.

## Discussion

The integrated dynamic Flux Balance Analysis (idFBA) framework presented here couples stoichiometric reconstructions of signaling, metabolic, and transcriptional regulatory networks with Flux Balance Analysis (FBA) to predict dynamic profiles of cellular phenotypes as a function of extracellular stimuli. Instantaneous inclusion of “slow” reactions in a time-delayed fashion accounted for network interactions occurring over a wide range of time scales. Previous approaches based on FBA have only addressed the coupling of regulatory structure with metabolic systems [29], which do not account for the effects of extracellular signaling cues on cellular phenotype.

The key features and results described here include: (1) an explicit accounting of the protein synthesis demands of a transcriptional regulatory network in the context of signaling and metabolic functions; (2) a quasi-steady-state description of cellular signaling events, readily interfaced with metabolic and regulatory networks; (3) similar dynamic profiles of phenotypic variables (e.g., biomass production) between the idFBA framework presented here and an explicit kinetic model; and (4) applicability of the idFBA framework to actual biological systems through an illustrative example using yeast osmoregulation and agreement with published values. To implement idFBA, the objective function for the underlying optimization problem included, for signaling networks, the reactions associated with the activation of transcription factors. The subsequent analysis resulted in “excluded reaction fluxes” (e.g., receptor internalization and protein degradation). These reactions were specified as “active” to denote their participation in the reaction network by imposing simple constraints (*v* = 0) on their counterparts, as described in “Conceptual Methods and Framework.”

Comparison with the detailed kinetic model validates the idFBA approach. Specifically, approximating the temporal progression of “slow” reactions in signaling, metabolic, and regulatory networks as steady-state constraints with time-delay and duration parameters provides acceptable predictions of the dynamic trends of a cell's phenotypic behavior. The primary motivation for comparing idFBA with a detailed kinetic model was to determine whether idFBA would yield comparable temporal behavior in spite of the inherent approximation it contained. Optimization-based approaches have provided accurate quantitative predictions of cellular growth [24]. However, signaling networks have not previously been modeled at a scale comparable to that of metabolic and regulatory networks [9]. Databases are increasingly available for signaling networks and efforts are ongoing to reconstruct larger, genome-scale signaling systems [32]. As this information becomes available, the idFBA framework can be applied to cellular systems and be coupled with experimental assays to generate quantitative hypotheses and assist in an iterative model-building process for deriving emergent properties of these systems.

The idFBA framework optimizes the system at the current time step, *t_current_*, according to the linear programming formulation of FBA (Equation 17).
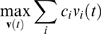
(17)Altering this framework to impose a multi-horizon formulation may facilitate the evaluation of different objective functions because the formulation naturally accounts for long-term effects of the calculated flux distribution at the current time step [25]. The multi-horizon formulation is shown in Equation 18, where *w_j_* is the weight associated with the objective after *T_j_* sample times.
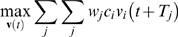
(18)


The main assumption of a multi-horizon formulation is that the flux distribution at *t_current_* is determined such that it maximizes a cellular objective within a certain future time period of interest. The resulting optimization problem, including a Boolean representation of the transcriptional regulatory network, becomes a multi-horizon, mixed-integer linear programming problem. Though the solution of such a problem needs further development for its scalability to large-scale systems [64], it may shed light on whether signal transduction at the current time step is optimally driven by a long-term objective. Currently, multi-stage optimization problems have been solved only for metabolic systems [25].

Recently, a method called Biological Objective Solution Search (BOSS) was developed for the inference of an objective function for a biological system from its underlying network stoichiometry as well as experimentally-measured flux distributions [54]. This method identifies objectives from experimental fluxes by defining a putative stoichiometric objective reaction, adding this reaction to the existing set of stoichiometric constraints, and maximizing it via linear programming. This new approach is capable of inferring the objective functions of metabolic networks, as well as metabolic and regulatory networks for which the objective is not well-characterized experimentally. Therefore, utilizing BOSS to identify objectives for the signaling, regulatory, and metabolic networks would facilitate the identification of an *in silico* flux distribution for the integrated system, a key step in the idFBA framework.

The fact that different reactions occur on different time scales (e.g., signaling reactions are usually fast whereas regulatory reactions are usually slow) is readily handled within the idFBA framework. Reactions with time constants of more than a unit time step are considered “slow”. However, identifying the optimal discretization of the time domain would facilitate a more accurate simulation for systems with multiple time-scales. Given typical rate constants, model reduction [15] and Monte Carlo sampling [65] techniques may help characterize representative time-scales of a given system as well.

As illustrated by the idFBA results for the prototypic integrated system and particularly the representative yeast module, the methodology and analyses afforded by this framework can provide insight into fundamental characteristics of biological systems, including network components and interactions. Evaluating how whole-cell systems respond to different perturbations, including modifications to environmental cues as well as intracellular reactions, can offer insights into disease mechanisms and possible therapeutic avenues. For example, assessing how genetic perturbations of signaling proteins affect the transcriptional program and metabolism of a cell is essential to fully appreciating the end-stage phenotypic effects of the perturbations on the whole cell. Furthermore, evaluating how modifications to an existing transcriptional regulatory program (e.g., altering the Boolean rules governing transcription of one or more genes) affect whole-cell behavior is essential in the design and engineering of metabolic systems. Such a complete picture of cellular response can drive accurate predictions of disease and drug discovery.

Additionally, unlike kinetic-based models and other similar approaches, the idFBA framework requires significantly fewer parameters and can facilitate an approximation of the dynamics of large-scale systems quickly and efficiently, given a stoichiometric network reconstruction. As has been hypothesized in the literature recently, our idFBA results support the theory that the structure of a network, rather than the detailed kinetic values that describe it, can drive the dynamics of its phenotype [66].

In conclusion, a novel technique called integrated dynamic Flux Balance Analysis (idFBA) has been developed to analyze integrated systems, and specifically to account for the interactions between signaling, metabolic, and transcriptional regulatory networks across many time scales. This approach facilitates the study of systemic effects of extracellular cues on cellular behavior in a quantitative manner. Additionally, the success of idFBA on a prototypic integrated system as well as a representative integrated yeast module serves as a benchmark for future analyses of integrated biochemical systems.

## Supporting Information

Text S1 - Prototypic Integrated System (0.06 MB PDF)

Text S2 - Representative Yeast Module (0.55 MB DOC)
