# Correction: Dynamic Analysis of Integrated Signaling, Metabolic, and Regulatory Networks

**DOI:** 10.1371/annotation/5594348b-de00-446a-bdd0-ec56e70b3553

**Published:** 2008-06-10

**Authors:** Jong Min Lee, Erwin P. Gianchandani, James A. Eddy, Jason A. Papin

The first author's name is incorrect in the article citation. It should appear as: Lee JM.

