# Supplementary material for: Dynamic Analysis of Integrated Signaling, Metabolic, and Regulatory Networks
Source: PLoS Comput Biol. 2008 May 23;4(5):e1000086. doi: 10.1371/journal.pcbi.1000086 (PMC2377155; doi:10.1371/journal.pcbi.1000086)
Supplement: Text S1 - Prototypic Integrated System — (0.06 MB PDF) [file pcbi.1000086.s001.pdf]

TEXT S1: PROTOTYPIC INTEGRATED SYSTEM

In an effort to investigate the **integrated dynamic Flux Balance Analysis** (**idFBA**) framework, a prototypic integrated network was assembled and evaluated. The prototypic integrated network is illustrated in Figure 3. Network components and component interactions were typical of those observed in published reconstructions of signaling, metabolic, and transcriptional regulatory networks. Here we summarize the kinetic relationships for the network that were used to implement the kinetic model described in the manuscript. Specifically, Tables S1.1 and S1.2 present the kinetic equations and rate constants for the prototypic signaling network, respectively; and Tables S1.3 and S1.4 the kinetic equations and rate constants for the prototypic metabolic network, respectively. In addition, the ordinary differential equations for the signaling, metabolic, and regulatory networks are Equations 1 through 53, 54 through 66, and 67 through 69, respectively.

REFERENCES

1. Lauffenburger DA, Linderman JJ (1993) Receptors: models for binding, trafficking, and signaling. New York: Oxford University Press. x, 365 p. p.
2. Schoeberl B, Eichler-Jonsson C, Gilles ED, Muller G (2002) Computational modeling of the dynamics of the MAP kinase cascade activated by surface and internalized EGF receptors. Nat Biotechnol 20: 370-375.
3. Klipp E, Nordlander B, Kruger R, Gennemark P, Hohmann S (2005) Integrative model of the response of yeast to osmotic shock. Nat Biotechnol 23: 975-982.

FIGURE CAPTIONS

Table S1.1. Kinetic equations of the prototypic signaling network.

Table S1.2. Rate constants for the prototypic signaling network.

Table S1.3. Kinetic equations of the prototypic metabolic network.

Table S1.4. Rate constants for the prototypic metabolic network.

Table S1.1. Kinetic equations of the prototypic signaling network.

Name	Reaction	Kinetic Equation	Description
v_1^{S1}	$[L_1]+[R_1]\rightarrow[L_1R_1]$	$k_1^{S1}[L_1][R_1]$	Binding
v_2^{S1}	$[L_1R_1]\rightarrow[L_1]+[R_1]$	$k_2^{S1}[L_1R_1]$	Dissociation
v_3^{S1}	$[L_1R_1]\rightarrow[L_1R_{1_{int}}]$	$k_3^{S1}[L_1R_1]$	Internalization
v_4^{S1}	$[L_1R_1]+[S_1]\rightarrow[L_1R_1\cdot S_1]$	$k_4^{S1}[L_1R_1][S_1]$	Association
v_5^{S1}	$[L_1R_1\cdot S_1]+[ATP]\rightarrow[L_1R_1\cdot S_1\cdot ATP]$	$k_5^{S1}[L_1R_1\cdot S_1][ATP]$	Association
v_6^{S1}	$[L_1R_1\cdot S_1\cdot ATP]\rightarrow[S_1p]+[ADP]+[L_1R_1]$	$k_6^{S1}[L_1R_1\cdot S_1\cdot ATP]$	Dissociation
v_7^{S1}	$[S_1p]+[T_1]\rightarrow[S_1p\cdot T_1]$	$k_7^{S1}[S_1p][T_1]$	Association
v_8^{S1}	$[S_1p\cdot T_1]+[ATP]\rightarrow[S_1p\cdot T_1\cdot ATP]$	$k_8^{S1}[S_1p\cdot T_1][ATP]$	Association
v_9^{S1}	$[S_1p\cdot T_1\cdot ATP]\rightarrow[S_1p]+[T_1p]+[ADP]$	$k_9^{S1}[S_1p\cdot T_1\cdot ATP]$	Dissociation
v_{10}^{S1}	$[S_1p]\rightarrow[S_1]+[H_2O]+[P_i]$	$k_{10}^{S1}[S_1p]$	Hydrolyzation
v_{11}^{S1}	$[T_1p]\rightarrow[T_1]+[H_2O]+[P_i]$	$k_{11}^{S1}[T_1p]$	Hydrolyzation
v_{12}^{S1}	$[S_1p]+[IS_1]\rightarrow[IS_1\cdot S_1p]$	$k_{12}^{S1}[S_1p][IS_1]$	Inhibition
v_{13}^{S1}	$[IS_1\cdot S_1p]\rightarrow[IS_1\cdot S_1p_{lys}]$	$k_{13}^{S1}[IS_1\cdot S_1p]$	Inhibition
$v_1^{S_1S_2}$	$[T_1]+[S_2p]\rightarrow[T_1\cdot S_2p]$	$k_1^{S_1S_2}[T_1][S_2p]$	Association
$v_2^{S_1S_2}$	$[T_1\cdot S_2p]+[ATP]\rightarrow[T_1\cdot S_2p\cdot ATP]$	$k_2^{S_1S_2}[T_1\cdot S_2p][ATP]$	Association
$v_3^{S_1S_2}$	$[T_1\cdot S_2p\cdot ATP]\rightarrow[T_1p]+[S_2p]+[ADP]$	$k_3^{S_1S_2}[T_1\cdot S_2p\cdot ATP]$	Dissociation
s_2	$[T_2]+[S_3p]\rightarrow[T_2\cdot S_3p]$	$k_1^{S_2S_3}[T_2][S_3p]$	Association
$v_2^{S_2S_3}$	$[T_2\cdot S_3p]+[ATP]\rightarrow[T_2\cdot S_3p\cdot ATP]$	$k_2^{S_2S_3}[T_2\cdot S_3p][ATP]$	Association
$v_3^{S_2S_3}$	$[T_2\cdot S_3p\cdot ATP]\rightarrow[T_2p]+[S_3p]+[ADP]$	$k_3^{S_2S_3}[T_2\cdot S_3p\cdot ATP]$	Dissociation
v_1^{S2}	$[L_2]+[R_2]\rightarrow[L_2R_2]$	$k_1^{S2}[L_2][R_2]$	Binding
v_2^{S2}	$[L_2R_2]\rightarrow[L_2]+[R_2]$	$k_2^{S2}[L_2R_2]$	Dissociation
v_3^{S2}	$[L_2R_2]\rightarrow[L_2R_{2_{int}}]$	$k_3^{S2}[L_2R_2]$	Internalization
v_4^{S2}	$[L_2R_2]+[S_2]\rightarrow[L_2R_2\cdot S_2]$	$k_4^{S2}[L_2R_2][S_2]$	Association
v_5^{S2}	$[L_2R_2\cdot S_2]+[ATP]\rightarrow[L_2R_2\cdot S_2\cdot ATP]$	$k_5^{S2}[L_2R_2\cdot S_2][ATP]$	Association
v_6^{S2}	$[L_2R_2\cdot S_2\cdot ATP]\rightarrow[S_2p]+[ADP]+[L_2R_2]$	$k_6^{S2}[L_2R_2\cdot S_2\cdot ATP]$	Dissociation
v_7^{S2}	$[S_2p]+[T_2]\rightarrow[S_2p\cdot T_2]$	$k_7^{S2}[S_2p][T_2]$	Association
v_8^{S2}	$[S_2p\cdot T_2]+[ATP]\rightarrow[S_2p\cdot T_2\cdot ATP]$	$k_8^{S2}[S_2p\cdot T_2][ATP]$	Association
v_9^{S2}	$[S_2p\cdot T_2\cdot ATP]\rightarrow[S_2p]+[T_2p]+[ADP]$	$k_9^{S2}[S_2p\cdot T_2\cdot ATP]$	Dissociation
v_{10}^{S2}	$[S_2p]\rightarrow[S_2]+[H_2O]+[P_i]$	$k_{10}^{S2}[S_2p]$	Hydrolyzation
v_{11}^{S2}	$[T_2p]\rightarrow[T_2]+[H_2O]+[P_i]$	$k_{11}^{S2}[T_2p]$	Hydrolyzation

v_{12}^{S2}	$[S_2 p]+[IS_2]\rightarrow[IS_2\cdot S_2 p]$	$k_{12}^{S2}[S_2 p][IS_2]$	Inhibition
v_{13}^{S2}	$[IS_2\cdot S_2 p]\rightarrow[IS_2\cdot S_2 p_{lys}]$	$k_{13}^{S2}[IS_2\cdot S_2 p]$	Inhibition
v_1^{S3}	$[L_3]+[R_3]\rightarrow[L_3 R_3]$	$k_1^{S3}[L_3][R_3]$	Binding
v_2^{S3}	$[L_3 R_3]\rightarrow[L_3]+[R_3]$	$k_2^{S3}[L_3 R_3]$	Dissociation
v_3^{S3}	$[L_3 R_3]\rightarrow[L_3 R_{3_{int}}]$	$k_3^{S3}[L_3 R_3]$	Internalization
v_4^{S3}	$[L_3 R_3]+[S_3]\rightarrow[L_3 R_3\cdot S_3]$	$k_4^{S3}[L_3 R_3][S_3]$	Association
v_5^{S3}	$[L_3 R_3\cdot S_3]+[ATP]\rightarrow[L_3 R_3\cdot S_3\cdot ATP]$	$k_5^{S3}[L_3 R_3\cdot S_3][ATP]$	Association
v_6^{S3}	$[L_3 R_3\cdot S_3\cdot ATP]\rightarrow[S_3 p]+[ADP]+[L_3 R_3]$	$k_6^{S3}[L_3 R_3\cdot S_3\cdot ATP]$	Dissociation
v_7^{S3}	$[S_3 p]+[T_3]\rightarrow[S_3 p\cdot T_3]$	$k_7^{S3}[S_3 p][T_3]$	Association
v_8^{S3}	$[S_3 p\cdot T_3]+[ATP]\rightarrow[S_3 p\cdot T_3\cdot ATP]$	$k_8^{S3}[S_3 p\cdot T_3][ATP]$	Association
v_9^{S3}	$[S_3 p\cdot T_3\cdot ATP]\rightarrow[S_3 p]+[T_3 p]+[ADP]$	$k_9^{S3}[S_3 p\cdot T_3\cdot ATP]$	Dissociation
v_{10}^{S3}	$[S_3 p]\rightarrow[S_3]+[H_2O]+[P_i]$	$k_{10}^{S3}[S_3 p]$	Hydrolyzation
v_{11}^{S3}	$[T_3 p]\rightarrow[T_3]+[H_2O]+[P_i]$	$k_{11}^{S3}[T_3 p]$	Hydrolyzation
v_{12}^{S3}	$[S_3 p]+[IS_3]\rightarrow[IS_3\cdot S_3 p]$	$k_{12}^{S3}[S_3 p][IS_3]$	Inhibition
v_{13}^{S3}	$[IS_3\cdot S_3 p]\rightarrow[IS_3\cdot S_3 p_{lys}]$	$k_{13}^{S3}[IS_3\cdot S_3 p]$	Inhibition

Table S1.2. Rate constants for the prototypic signaling network.

Parameter	Value	Unit	Reference	Parameter	Value	Unit	Reference
$k_1^{S_1}$	3.0	$[\frac{1}{\mu M \cdot s}]$	[1], Table 2-1	$k_2^{S_1}$	0.002	$[\frac{1}{s}]$	[1], Table 2-1
$k_3^{S_1}$	0.001	$[\frac{1}{s}]$	[1], Fig. 3-7	$k_4^{S_1}$	10	$[\frac{1}{\mu M \cdot s}]$	[2], v_2
$k_5^{S_1}$	3.3	$[\frac{1}{\mu M \cdot s}]$	[2], v_2	$k_6^{S_1}$	2.9	$[\frac{1}{s}]$	[2], v_{47}
$k_7^{S_1}$	6.7	$[\frac{1}{\mu M \cdot s}]$	[2], v_2	$k_8^{S_1}$	2.5	$[\frac{1}{\mu M \cdot s}]$	[2], v_2
$k_9^{S_1}$	5.0	$[\frac{1}{s}]$	[2], v_{12}	$k_{10}^{S_1}$	0.001	$[\frac{1}{s}]$	[3], k_{-i}^{MAP}
$k_{11}^{S_1}$	0.001	$[\frac{1}{s}]$	[3], k_{-i}^{MAP}	$k_{12}^{S_1}$	0.001	$[\frac{1}{\mu M \cdot s}]$	[1]
$k_{13}^{S_1}$	0.003	$[\frac{1}{s}]$	[1], Table 6-2	$k_1^{S_1S_2}$	4.2	$[\frac{1}{\mu M \cdot s}]$	[2], v_2
$k_2^{S_1S_2}$	5.0	$[\frac{1}{\mu M \cdot s}]$		$k_3^{S_1S_2}$	1.0	$[\frac{1}{s}]$	
$k_1^{S_2S_3}$	5.0	$[\frac{1}{\mu M \cdot s}]$	[2], v_2	$k_2^{S_2S_3}$	4.0	$[\frac{1}{\mu M \cdot s}]$	
$k_3^{S_2S_3}$	1.0	$[\frac{1}{s}]$					
$k_1^{S_2}$	2.85	$[\frac{1}{\mu M \cdot s}]$	[1], Table 2-1	$k_2^{S_2}$	0.0019	$[\frac{1}{s}]$	[1], Table 2-1
$k_3^{S_2}$	0.00095	$[\frac{1}{s}]$	[1], Fig. 3-7	$k_4^{S_2}$	9.5	$[\frac{1}{\mu M \cdot s}]$	[2], v_2
$k_5^{S_2}$	3.1	$[\frac{1}{\mu M \cdot s}]$	[2], v_2	$k_6^{S_2}$	2.7	$[\frac{1}{s}]$	[2], v_{47}
$k_7^{S_2}$	6.3	$[\frac{1}{\mu M \cdot s}]$	[2], v_2	$k_8^{S_2}$	2.3	$[\frac{1}{\mu M \cdot s}]$	[2], v_2
$k_9^{S_2}$	4.75	$[\frac{1}{s}]$	[2], v_{12}	$k_{10}^{S_2}$	0.00095	$[\frac{1}{s}]$	[3], k_{-i}^{MAP}
$k_{11}^{S_2}$	0.00095	$[\frac{1}{s}]$	[3], k_{-i}^{MAP}	$k_{12}^{S_2}$	0.00095	$[\frac{1}{\mu M \cdot s}]$	[1]
$k_{13}^{S_2}$	0.0028	$[\frac{1}{s}]$	[1], Table 6-2				
$k_1^{S_3}$	3.15	$[\frac{1}{\mu M \cdot s}]$	[1], Table 2-1	$k_2^{S_3}$	0.0021	$[\frac{1}{s}]$	[1], Table 2-1

k_3^{S3}	0.0011	$[\frac{1}{s}]$	[1], Fig. 3-7	k_4^{S3}	10.5	$[\frac{1}{\mu M \cdot s}]$	[2], v_2
k_5^{S3}	3.5	$[\frac{1}{\mu M \cdot s}]$	[2], v_2	k_6^{S3}	3.1	$[\frac{1}{s}]$	[2], v_{47}
k_7^{S3}	7.0	$[\frac{1}{\mu M \cdot s}]$	[2], v_2	k_8^{S3}	2.6	$[\frac{1}{\mu M \cdot s}]$	[2], v_2
k_9^{S3}	5.25	$[\frac{1}{s}]$	[2], v_{12}	k_{10}^{S3}	0.0011	$[\frac{1}{s}]$	[3], k_{-i}^{MAP}
k_{11}^{S3}	0.0012	$[\frac{1}{s}]$	[3], k_{-i}^{MAP}	k_{12}^{S3}	0.0012	$[\frac{1}{\mu M \cdot s}]$	[1]
k_{13}^{S3}	0.0032	$[\frac{1}{s}]$	[1], Table 6-2				

Ordinary differential equations for the prototypic signaling network:

$$\frac{d}{dt}[L_1] = -v_1^{S1} + v_2^{S1} \tag{1}$$

$$\frac{d}{dt}[R_1] = -v_1^{S1} + v_2^{S1} + v_{reg} - k_{deg}[R_1] \tag{2}$$

$$\frac{d}{dt}[L_1R_1] = v_1^{S1} - v_2^{S1} - v_3^{S1} - v_4^{S1} + v_6^{S1} \tag{3}$$

$$\frac{d}{dt}[L_1R_{1_{int}}] = v_3^{S1} \tag{4}$$

$$\frac{d}{dt}[S_1] = -v_4^{S1} + v_{10}^{S1} + v_{reg} - k_{deg}[S_1] \tag{5}$$

$$\frac{d}{dt}[L_1R_1 \cdot S_1] = v_4^{S1} - v_5^{S1} \tag{6}$$

$$\frac{d}{dt}[L_1R_1 \cdot S_1 \cdot ATP] = v_5^{S1} - v_6^{S1} \tag{7}$$

$$\frac{d}{dt}[S_1p] = v_6^{S1} - v_7^{S1} + v_9^{S1} - v_{10}^{S1} - v_{12}^{S1} \tag{8}$$

$$\frac{d}{dt}[T_1] = -v_7^{S1} + v_{11}^{S1} - v_1^{S1S2} + v_{reg} - k_{deg}[T_1] \tag{9}$$

$$\frac{d}{dt}[T_1p] = v_9^{S1} - v_{11}^{S1} + v_3^{S1S2} \tag{10}$$

$$\frac{d}{dt}[S_1p \cdot T_1] = v_7^{S1} - v_8^{S1} \tag{11}$$

$$\frac{d}{dt}[S_1p \cdot T_1 \cdot ATP] = v_8^{S1} - v_9^{S1} \tag{12}$$

$$\frac{d}{dt}[IS_1] = -v_{12}^{S1} + v_{reg} - k_{deg}[IS_1] \tag{13}$$

$$\frac{d}{dt}[IS_1 \cdot S_1p] = v_{12}^{S1} - v_{13}^{S1} \tag{14}$$

$$\frac{d}{dt}[IS_1 \cdot S_1p_{lys}] = v_{13}^{S1} \tag{15}$$

$$\frac{d}{dt}[L_2] = -v_1^{S2} + v_2^{S2} \tag{16}$$

$$\frac{d}{dt}[R_2] = -v_1^{S_2} + v_2^{S_2} + v_{reg} - k_{deg}[R_2] \qquad (17)$$

$$\frac{d}{dt}[L_2R_2] = v_1^{S_2} - v_2^{S_2} - v_3^{S_2} - v_4^{S_2} + v_6^{S_2} \qquad (18)$$

$$\frac{d}{dt}[L_2R_{2_{int}}] = v_3^{S_2} \qquad (19)$$

$$\frac{d}{dt}[S_2] = -v_4^{S_2} + v_{10}^{S_2} + v_{reg} - k_{deg}[S_2] \qquad (20)$$

$$\frac{d}{dt}[L_2R_2 \cdot S_2] = v_4^{S_2} - v_5^{S_2} \qquad (21)$$

$$\frac{d}{dt}[L_2R_2 \cdot S_2 \cdot ATP] = v_5^{S_2} - v_6^{S_2} \qquad (22)$$

$$\frac{d}{dt}[S_2 p] = v_6^{S_2} - v_7^{S_2} + v_9^{S_2} - v_{10}^{S_2} - v_{12}^{S_2} - v_1^{S_1 S_2} + v_3^{S_1 S_2} \qquad (23)$$

$$\frac{d}{dt}[T_2] = -v_7^{S_2} + v_{11}^{S_2} + v_{reg} - k_{deg}[T_2] \qquad (24)$$

$$\frac{d}{dt}[T_2 p] = v_9^{S_2} - v_{11}^{S_2} \qquad (25)$$

$$\frac{d}{dt}[S_2 p \cdot T_2] = v_7^{S_2} - v_8^{S_2} \qquad (26)$$

$$\frac{d}{dt}[S_2 p \cdot T_2 \cdot ATP] = v_8^{S_2} - v_9^{S_2} \qquad (27)$$

$$\frac{d}{dt}[IS_2] = -v_{12}^{S_2} + v_{reg} - k_{deg}[IS_2] \qquad (28)$$

$$\frac{d}{dt}[IS_2 \cdot S_2 p] = v_{12}^{S_2} - v_{13}^{S_2} \qquad (29)$$

$$\frac{d}{dt}[IS_2 \cdot S_2 p_{lys}] = v_{13}^{S_2} \qquad (30)$$

$$\frac{d}{dt}[L_3] = -v_1^{S_3} + v_2^{S_3} \qquad (31)$$

$$\frac{d}{dt}[R_3] = -v_1^{S_3} + v_2^{S_3} + v_{reg} - k_{deg}[R_3] \qquad (32)$$

$$\frac{d}{dt}[L_3R_3] = v_1^{S_3} - v_2^{S_3} - v_3^{S_3} - v_4^{S_3} + v_6^{S_3} \qquad (33)$$

$$\frac{d}{dt}[L_3R_{3_{int}}] = v_3^{S3} \tag{34}$$

$$\frac{d}{dt}[S_3] = -v_4^{S3} + v_{10}^{S3} + v_{reg} - k_{deg}[S_3] \tag{35}$$

$$\frac{d}{dt}[L_3R_3 \cdot S_3] = v_4^{S3} - v_5^{S3} \tag{36}$$

$$\frac{d}{dt}[L_3R_3 \cdot S_3 \cdot ATP] = v_5^{S3} - v_6^{S3} \tag{37}$$

$$\frac{d}{dt}[S_3p] = v_6^{S3} - v_7^{S3} + v_9^{S3} - v_{10}^{S3} - v_{12}^{S3} - v_1^{S_2S_3} + v_3^{S_2S_3} \tag{38}$$

$$\frac{d}{dt}[T_3] = -v_7^{S3} + v_{11}^{S3} + v_{reg} - k_{deg}[T_3] \tag{39}$$

$$\frac{d}{dt}[T_3p] = v_9^{S3} - v_{11}^{S3} \tag{40}$$

$$\frac{d}{dt}[S_3p \cdot T_3] = v_7^{S3} - v_8^{S3} \tag{41}$$

$$\frac{d}{dt}[S_3p \cdot T_3 \cdot ATP] = v_8^{S3} - v_9^{S3} \tag{42}$$

$$\frac{d}{dt}[IS_3] = -v_{12}^{S3} + v_{reg} - k_{deg}[IS_3] \tag{43}$$

$$\frac{d}{dt}[IS_3 \cdot S_3p] = v_{12}^{S3} - v_{13}^{S3} \tag{44}$$

$$\frac{d}{dt}[IS_3 \cdot S_3p_{lys}] = v_{13}^{S3} \tag{45}$$

$$\frac{d}{dt}[T_1 \cdot S_2p] = v_1^{S_1S_2} - v_2^{S_1S_2} \tag{46}$$

$$\frac{d}{dt}[T_1 \cdot S_2p \cdot ATP] = v_2^{S_1S_2} - v_3^{S_1S_2} \tag{47}$$

$$\frac{d}{dt}[T_2 \cdot S_3p] = v_1^{S_2S_3} - v_2^{S_2S_3} \tag{48}$$

$$\frac{d}{dt}[T_2 \cdot S_3p \cdot ATP] = v_2^{S_2S_3} - v_3^{S_2S_3} \tag{49}$$

$$\frac{d}{dt}[H_2O] = v_{10}^{S1} + v_{11}^{S1} + v_{10}^{S2} + v_{11}^{S2} + v_{10}^{S3} + v_{11}^{S3} \tag{50}$$

$$\frac{d}{dt}[P_i] = v_{10}^{S_1} + v_{11}^{S_1} + v_{10}^{S_2} + v_{11}^{S_2} + v_{10}^{S_3} + v_{11}^{S_3} \tag{51}$$

$$\frac{d}{dt}[ATP] = \frac{-v_5^{S_1} - v_8^{S_1} - v_5^{S_2} - v_8^{S_2} - v_5^{S_3} - v_8^{S_3} - v_2^{S_1S_2} - v_2^{S_2S_3}}{1000} - v_2^m - v_3^m + 2v_6^m - 5v_8^m - v_9^m + 3v_{11}^m - v_{13}^m \tag{52}$$

$$\frac{d}{dt}[ADP] = \frac{v_6^{S_1} + v_9^{S_1} + v_6^{S_2} + v_9^{S_2} + v_6^{S_3} + v_9^{S_3} + v_3^{S_1S_2} + v_3^{S_2S_3}}{1000} + v_2^m + v_3^m - 2v_6^m + 5v_8^m + v_9^m - 3v_{11}^m + v_{13}^m \tag{53}$$

Table S1.3. Kinetic equations of the prototypic metabolic network.

Name	Reaction	Kinetic Equation
v_1^m	$[Carbon] \rightarrow [A]$	$S_u \cdot [Biomass]$
v_2^m	$[A]+[ATP] \rightarrow [B]+[ADP]$	$\dfrac{k_2^m}{1+\dfrac{k_{21}^m}{[ATP]}\left(1+\dfrac{[ADP]}{k_{22}^m}\right)+\dfrac{k_{23}^m}{[A]}+\dfrac{k_{21}^m}{[ATP]}\cdot\dfrac{k_{23}^m}{[A]}\cdot\left(1+\dfrac{[ADP]}{k_{22}^m}\right)}$
v_3^m	$[B]+[ATP] \rightarrow [C]+[ADP]$	$k_3^m \dfrac{[ATP]}{k_{31}^m+[ATP]}\dfrac{[B]}{k_{32}^m+[B]}$
v_4^m	$[C] \rightarrow [D]+[E]$	$k_4^m \dfrac{\left([C]-\dfrac{[D][E]}{K_4^m}\right)}{k_{41}^m+[C]+\dfrac{k_{42}^m[D]}{K_4^m k_{43}^m}+\dfrac{k_{44}^m[E]}{K_4^m k_{43}^m}+\dfrac{[C][D]}{K_{41}^m}+\dfrac{[D][E]}{K_4^m k_{43}^m}}$
v_5^m	$[D] \rightarrow [E]$	$-k_5^m \dfrac{[E]-\dfrac{[D]}{K_5^m}}{k_{51}^m\left(1+\dfrac{[D]}{k_{52}^m}\right)+[E]}$
v_6^m	$[D]+2[ADP]+[NAD] \rightarrow$ $[G]+2[ATP]+[NADH]$	$k_6^m[D][NAD][ADP]$
v_7^m	$[G]+[NADH] \rightarrow [H_2]+[NAD]$	$k_7^m \dfrac{[G]}{k_{71}^m+[G]}\dfrac{[NADH]}{k_{72}^m+[NADH]}$
v_8^m	$1.5[F]+10[G]+2[H_1]+2[H_2]$ $+5[ATP] \rightarrow [Biomass]+5[ADP]$	$k_8^m \dfrac{[G]}{k_{81}^m+[G]}\dfrac{[ATP]}{k_{82}^m+[ATP]}\dfrac{[F]}{k_{83}^m+[F]}\dfrac{[H_1]}{k_{84}^m+[H_1]}\dfrac{[H_2]}{k_{85}^m+[H_2]}$
v_9^m	$0.8[B]+[ATP] \rightarrow [H_1]+[ADP]$	$k_9^m \dfrac{[B]}{k_{91}^m+[B]}\dfrac{[ATP]}{k_{92}^m+[ATP]}$
v_{10}^m	$[E]+[NADH] \rightarrow [F]+[NAD]$	$k_{10}^m \dfrac{[E]}{k_{101}^m+[E]}\dfrac{[NADH]}{k_{102}^m+[NADH]}$
v_{11}^m	$3[ADP]+[NADH] \rightarrow$ $3[ATP]+[NAD]$	$k_{11}^m \dfrac{[NADH][ADP]}{k_{111}^m+[ADP]}$
v_{12}^m	$[NAD] \rightarrow [NADH]$	$k_{12}^m[NAD]$
v_{13}^m	$[ATP] \rightarrow [ADP]$	$k_{13}^m \dfrac{[ATP]}{k_{131}^m+[ATP]}$

Table S1.4. Rate constants of the prototypic metabolic network.

Parameter	Value	Unit	Reference	Parameter	Value	Unit	Reference
k_2^m	1.77	$[\frac{mM}{s}]$	[3], Eq. 5.15	k_{21}^m	0.2	$[mM]$	[3], Eq. 5.15
k_{22}^m	1.2	$[mM]$	[3], Eq. 5.15	k_{23}^m	0.2	$[mM]$	[3], Eq. 5.15
k_3^m	0.895	$[\frac{mM}{s}]$	[3], Eq. 5.16	k_{31}^m	0.01	$[mM]$	[3], Eq. 5.16
k_{32}^m	0.012	$[mM]$	[3], Eq. 5.16	k_4^m	1867.4	$[\frac{mM}{s}]$	[3], Eq. 5.17
K_4^m	0.81	$[mM]$	[3], Eq. 5.17	k_{41}^m	0.054	$[mM]$	[3], Eq. 5.17
k_{42}^m	2.0	$[mM]$	[3], Eq. 5.17	k_{43}^m	5.0		[3], Eq. 5.17
k_{44}^m	2.0	$[mM]$	[3], Eq. 5.17	K_{41}^m	10.0	$[mM]$	[3], Eq. 5.17
k_5^m	190	$[\frac{mM}{s}]$	[3], Eq. 5.18	K_5^m	0.045		[3], Eq. 5.18
k_{51}^m	0.38	$[mM]$	[3], Eq. 5.18	k_{52}^m	0.064	$[mM]$	[3], Eq. 5.18
k_6^m	45.127	$[\frac{1}{mM^2 s}]$	[3], Eq. 5.19	k_7^m	1.5	$[\frac{mM}{s}]$	[3], Eq. 5.21
k_{71}^m	1.2	$[mM]$	[3], Eq. 5.21	k_{72}^m	0.6	$[mM]$	[3], Eq. 5.21
k_8^m	0.8090	$[\frac{g}{l \cdot s}]$	[3], Eq. 5.22	k_{81}^m	0.001	$[mM]$	[3], Eq. 5.22
k_{82}^m	0.5	$[mM]$	[3], Eq. 5.22	k_{83}^m	0.01	$[mM]$	[3], Eq. 5.22
k_{84}^m	0.05	$[mM]$	[3], Eq. 5.22	k_{85}^m	1.0	$[mM]$	[3], Eq. 5.22
k_9^m	0.4	$[\frac{mM}{s}]$	[3], Eq. 5.23	k_{91}^m	0.01	$[mM]$	[3], Eq. 5.23
k_{92}^m	0.01	$[mM]$	[3], Eq. 5.23	k_{10}^m	7.15	$[\frac{1}{mM \cdot s}]$	[3], Eq. 5.24
k_{101}^m	0.1	$[mM]$	[3], Eq. 5.24	k_{102}^m	0.6	$[mM]$	[3], Eq. 5.24
k_{11}^m	384	$[s^{-1}]$	[3], Eq. 5.27	k_{111}^m	0.42	$[mM]$	[3], Eq. 5.27
k_{12}^m	1.12	$[s^{-1}]$	[3], Eq. 5.28	k_{13}^m	99.8	$[\frac{mM}{s}]$	[3], Eq. 5.29
k_{131}^m	5.0	$[mM]$	[3], Eq. 5.29				

Ordinary differential equations for the prototypic metabolic network:

$$\frac{d}{dt}[A] = v_1^m - v_2^m \tag{54}$$

$$\frac{d}{dt}[B] = v_2^m - v_3^m - 0.8v_9^m \tag{55}$$

$$\frac{d}{dt}[C] = v_3^m - v_4^m \tag{56}$$

$$\frac{d}{dt}[D] = v_4^m - v_5^m - v_6^m \tag{57}$$

$$\frac{d}{dt}[E] = v_4^m + v_5^m - v_{10}^m \tag{58}$$

$$\frac{d}{dt}[F] = v_{10}^m - 1.5v_8^m \tag{59}$$

$$\frac{d}{dt}[G] = v_6^m - v_7^m - 10v_8^m \tag{60}$$

$$\frac{d}{dt}[H_1] = v_9^m - 2v_8^m - 0.371v_{reg} \tag{61}$$

$$\frac{d}{dt}[H_2] = v_7^m - 2v_8^m - 0.556v_{reg} \tag{62}$$

$$\frac{d}{dt}[NADH] = v_6^m - v_7^m - v_{10}^m - v_{11}^m + v_{12}^m \tag{63}$$

$$\frac{d}{dt}[NAD] = -\frac{d}{dt}[NADH] \tag{64}$$

$$\frac{d}{dt}[Biomass] = v_8^m \tag{65}$$

$$\frac{d}{dt}[Carbon] = -v_1^m \tag{66}$$

Ordinary differential equations for the prototypic regulatory network:

$$v_{reg} = k_{poly}[H_1][H_2], k_{poly} = 10^{-4}\left[\frac{1}{\mu M \cdot s}\right] \tag{67}$$

$$v_{deg} = k_{deg}[Protein], k_{deg} = 10^{-3}\left[\frac{1}{s}\right] \tag{68}$$

$$\frac{d}{dt}[Protein] = v_{reg} - v_{deg} \tag{69}$$
