# Supplementary material for: Dynamic Analysis of Integrated Signaling, Metabolic, and Regulatory Networks
Source: PLoS Comput Biol. 2008 May 23;4(5):e1000086. doi: 10.1371/journal.pcbi.1000086 (PMC2377155; doi:10.1371/journal.pcbi.1000086)
Supplement: Text S2 - Representative Yeast Module — (0.55 MB DOC) [file pcbi.1000086.s002.doc]

**TEXT S2: REPRESENTATIVE YEAST MODULE**

In an effort to assess the applicability of the **integrated dynamic Flux Balance Analysis** (**idFBA**) framework to actual biological systems, a representative integrated module from the prototypic single-cell eukaryote *Saccharomyces cerevisiae* was selected for interrogation. This module was comprised of key aspects of yeast osmoregulation, as described further in the manuscript. Specifically, the signaling, metabolic, and regulatory processes associated with the high-osmolarity glycerol response (HOG) mitogen-activated protein (MAP) kinase pathway were assimilated from existing literature. Here we summarize the kinetic relationships for the network that were used to implement the kinetic model described in the manuscript. Specifically, Tables S2.1 and S2.2 present the kinetic equations and rate constants for the representative signaling network, respectively; and Tables S2.3 and S2.4 the kinetic equations and rate constants for the representative metabolic network, respectively. In addition, the ordinary differential equations for the signaling, metabolic, and regulatory networks are Equations 1 through 33, 34 through 49, and 50 through 56, respectively.

**REFERENCES**

1. Lauffenburger DA, Linderman JJ (1993) Receptors: models for binding, trafficking, and signaling. New York: Oxford University Press. x, 365 p. p.

2. Klipp E, Nordlander B, Kruger R, Gennemark P, Hohmann S (2005) Integrative model of the response of yeast to osmotic shock. Nat Biotechnol 23: 975-982.

**FIGURE CAPTIONS**

**Table S2.1.** Kinetic equations of the representative signaling network.

**Table S2.2.** Rate constants for the representative signaling network.

**Table S2.3.** Kinetic equations of the representative metabolic network.

**Table S2.4.** Rate constants for the representative metabolic network.

**Table S2.1.** Kinetic equations of the representative signaling network.

| **Name** | **Reaction** | **Kinetic Equation** |
| --- | --- | --- |
| $v_{1}^{s}$ | $\left[\text{Sln1p}_{\text{c}}\right]+\left[\text{H}_{2}\text{O}_{\text{c}}\right]+\left[\text{osmotic\_stress}_{\text{e}}\right]\to$ $\left[\text{Sln1}_{\text{c}}\right]+\left[\text{P}_{\text{c}}\right]+\left[\text{H}^{+}_{\text{c}}\right]$ | $k_{1}^{s}\cdot\left[\text{Sln1p}_{\text{c}}\right]\cdot\left[\text{H}_{2}\text{O}_{\text{c}}\right]\cdot\left[\text{osmotic\_stress}_{\text{e}}\right]$ |
| $v_{2}^{s}$ | $\left[\text{Sln1p}_{\text{c}}\right]+\left[\text{Ypd1}_{\text{c}}\right]+\left[\text{ATP}_{\text{c}}\right]\to$ $\left[\text{Sln1}_{\text{c}}\right]+\left[\text{Ypd1p}_{\text{c}}\right]+\left[\text{ADP}_{\text{c}}\right]$ | $k_{2}^{s}\cdot\left[\text{Ypd1}_{\text{c}}\right]\cdot\left[\text{ATP}_{\text{c}}\right]$ |
| $v_{3}^{s}$ | $\left[\text{Sln1}_{\text{c}}\right]+\left[\text{Ssk1}_{\text{c}}\right]\to\left[\text{Sln1}_{\text{ex}}\right]+\left[\text{Ssk1\_osmosis}_{\text{c}}\right]$ | $k_{3}^{s}\cdot\left[\text{Sln1}_{\text{c}}\right]\cdot\left[\text{Ssk1}_{\text{c}}\right]$ |
| $v_{4}^{s}$ | $\left[\text{Ypd1p}_{\text{c}}\right]+\left[\text{Ssk1}_{\text{c}}\right]\to\left[\text{Ypd1}_{\text{c}}\right]+\left[\text{Ssk1p}_{\text{c}}\right]$ | $k_{4}^{s}\cdot\left[\text{Ypd1p}_{\text{c}}\right]\cdot\left[\text{Ssk1}_{\text{c}}\right]$ |
| $v_{5}^{s}$ | $\left[\text{Ssk1\_osmosis}_{\text{c}}\right]+\left[\text{Ssk2}_{\text{c}}\right]+\left[\text{ATP}_{\text{c}}\right]\to$ $\left[\text{Ssk1\_osmosis}_{\text{ex}}\right]+\left[\text{Ssk2p}_{\text{c}}\right]+\left[\text{ADP}_{\text{c}}\right]$ | $k_{5}^{s}\cdot\left[\text{Ssk1\_osmosis}_{\text{c}}\right]\cdot\left[\text{Ssk2}_{\text{c}}\right]\cdot\left[\text{ATP}_{\text{c}}\right]$ |
| $v_{6}^{s}$ | $\left[\text{Ssk2p}_{\text{c}}\right]+\left[\text{Pbs2}_{\text{c}}\right]+2\left[\text{ATP}_{\text{c}}\right]\to$ $\left[\left(\text{Ssk2p}\cdot\text{Pbs2pp}\right)_{\text{c}}\right]+2\left[\text{ADP}_{\text{c}}\right]$ | $k_{6}^{s}\cdot\left[\text{Ssk2p}_{\text{c}}\right]\cdot\left[\text{Pbs2}_{\text{c}}\right]\cdot2\left[\text{ATP}_{\text{c}}\right]$ |
| $v_{7}^{s}$ | $\left[\left(\text{Ssk2p}\cdot\text{Pbs2pp}\right)_{\text{c}}\right]+\left[\text{Hog1}_{\text{c}}\right]+2\left[\text{ATP}_{\text{c}}\right]\to$ $\left[\left(\text{Ssk2p}\cdot\text{Pbs2pp}\cdot\text{Hog1pp}\right)_{\text{c}}\right]+2\left[\text{ADP}_{\text{c}}\right]$ | $k_{7}^{s}\cdot\left[\left(\text{Ssk2p}\cdot\text{Pbs2pp}\right)_{\text{c}}\right]\cdot\left[\text{Hog1}_{\text{c}}\right]\cdot2\left[\text{ATP}_{\text{c}}\right]$ |
| $v_{8}^{s}$ | $\left[\left(\text{Ssk2p}\cdot\text{Pbs2pp}\cdot\text{Hog1pp}\right)_{\text{c}}\right]\to$ $\left[\left(\text{Ssk2p}\cdot\text{Pbs2pp}\right)_{\text{ex}}\right]+\left[\text{Hog1pp}_{\text{c}}\right]$ | $k_{8}^{s}\cdot\left[\left(\text{Ssk2p}\cdot\text{Pbs2pp}\cdot\text{Hog1pp}\right)_{\text{c}}\right]$ |
| $v_{9}^{s}$ | $\left[\text{Ssk1\_osmosis}_{\text{c}}\right]+\left[\text{Ssk22}_{\text{c}}\right]+\left[\text{ATP}_{\text{c}}\right]\to$ $\left[\text{Ssk1\_osmosis}_{\text{ex}}\right]+\left[\text{Ssk22p}_{\text{c}}\right]+\left[\text{ADP}_{\text{c}}\right]$ | $k_{9}^{s}\cdot\left[\text{Ssk1\_osmosis}_{\text{c}}\right]\cdot\left[\text{Ssk22}_{\text{c}}\right]\cdot\left[\text{ATP}_{\text{c}}\right]$ |
| $v_{10}^{s}$ | $\left[\text{Ssk22p}_{\text{c}}\right]+\left[\text{Pbs2}_{\text{c}}\right]+2\left[\text{ATP}_{\text{c}}\right]\to$ $\left[\left(\text{Ssk22p}\cdot\text{Pbs2pp}\right)_{\text{c}}\right]+2\left[\text{ADP}_{\text{c}}\right]$ | $k_{10}^{s}\cdot\left[\text{Ssk22p}_{\text{c}}\right]\cdot\left[\text{Pbs2}_{\text{c}}\right]\cdot2\left[\text{ATP}_{\text{c}}\right]$ |
| $v_{11}^{s}$ | $\left[\left(\text{Ssk22p}\cdot\text{Pbs2pp}\right)_{\text{c}}\right]+\left[\text{Hog1}_{\text{c}}\right]+2\left[\text{ATP}_{\text{c}}\right]\to$ $\left[\left(\text{Ssk22p}\cdot\text{Pbs2pp}\cdot\text{Hog1pp}\right)_{\text{c}}\right]+2\left[\text{ADP}_{\text{c}}\right]$ | $k_{11}^{s}\cdot\left[\left(\text{Ssk22p}\cdot\text{Pbs2pp}\right)_{\text{c}}\right]\cdot\left[\text{Hog1}_{\text{c}}\right]\cdot2\left[\text{ATP}_{\text{c}}\right]$ |
| $v_{12}^{s}$ | $\left[\left(\text{Ssk22p}\cdot\text{Pbs2pp}\cdot\text{Hog1pp}\right)_{\text{c}}\right]\to$ $\left[\left(\text{Ssk22p}\cdot\text{Pbs2pp}\right)_{\text{ex}}\right]+\left[\text{Hog1pp}_{\text{c}}\right]$ | $k_{12}^{s}\cdot\left[\left(\text{Ssk22p}\cdot\text{Pbs2pp}\cdot\text{Hog1pp}\right)_{\text{c}}\right]$ |
| $v_{13}^{s}$ | $\left[\text{Hog1pp}_{\text{c}}\right]\to\left[\text{Hog1pp}_{\text{n}}\right]$ | $k_{13}^{s}\cdot\left[\text{Hog1pp}_{\text{c}}\right]$ |
| $v_{14}^{s}$ | $\left[\text{Hog1pp}_{\text{n}}\right]+\left[\text{Hot1}_{\text{n}}\right]+2\left[\text{ATP}_{\text{n}}\right]\to$ $\left[\left(\text{Hog1pp}\cdot\text{Hot1pp}\right)_{\text{n}}\right]+2\left[\text{ADP}_{\text{n}}\right]$ | $k_{14}^{s}\cdot\left[\text{Hog1pp}_{\text{n}}\right]\cdot\left[\text{Hot1}_{\text{n}}\right]\cdot2\left[\text{ATP}_{\text{n}}\right]$ |
| $v_{15}^{s}$ | $\left[\text{ATP}_{\text{c}}\right]\to\left[\text{ATP}_{\text{n}}\right]$ | $k_{15}^{s}\cdot\left[\text{ATP}_{\text{c}}\right]$ |
| $v_{16}^{s}$ | $\left[\text{ADP}_{\text{n}}\right]\to\left[\text{ADP}_{\text{c}}\right]$ | $k_{16}^{s}\cdot\left[\text{ADP}_{\text{n}}\right]$ |

**Table S.2.** Rate constants for the representative signaling network.

| **Parameter** | **Value** | **Unit** | **Reference** | **Parameter** | **Value** | **Unit** | **Reference** |
| --- | --- | --- | --- | --- | --- | --- | --- |
| $k_{1}^{s}$ | 3.0 | $\text{[}\frac{1}{\mu M\cdot s}]$ | [1] | $k_{2}^{s}$ | 50 | $\text{[}\frac{1}{\mu M\cdot s}]$ | [2] |
| $k_{3}^{s}$ | 50 | $\text{[}\frac{1}{\mu M\cdot s}]$ | [2] | $k_{4}^{s}$ | 50 | $\text{[}\frac{1}{\mu M\cdot s}]$ | [2] |
| $k_{5}^{s}$ | 50 | $\text{[}\frac{1}{\mu M\cdot s}]$ | [2] | $k_{6}^{s}$ | 50 | $\text{[}\frac{1}{\mu M\cdot s}]$ | [2] |
| $k_{7}^{s}$ | 50 | $\text{[}\frac{1}{\mu M\cdot s}]$ | [2] | $k_{8}^{s}$ | 50 | $\text{[}\frac{1}{\mu M\cdot s}]$ | [2] |
| $k_{9}^{s}$ | 50 | $\text{[}\frac{1}{\mu M\cdot s}]$ | [2] | $k_{10}^{s}$ | 50 | $\text{[}\frac{1}{\mu M\cdot s}]$ | [2] |
| $k_{11}^{s}$ | 50 | $\text{[}\frac{1}{\mu M\cdot s}]$ | [2] | $k_{12}^{s}$ | 50 | $\text{[}\frac{1}{\mu M\cdot s}]$ | [2] |
| $k_{13}^{s}$ | 50 | $\text{[}\frac{1}{\mu M\cdot s}]$ | [2] | $k_{14}^{s}$ | 10.925 | $\text{[}\frac{1}{\mu M\cdot s}]$ | [2] |
| $k_{15}^{s}$ | 50 | $\text{[}\frac{1}{\mu M\cdot s}]$ | [2] | $k_{16}^{s}$ | 50 | $\text{[}\frac{1}{\mu M\cdot s}]$ | [2] |

**Ordinary differential equations for the representative signaling network:**

$\frac{d}{dt}\left[\text{ADP}_{\text{c}}\right]=v_{2}^{s}+v_{5}^{s}+2v_{6}^{s}+2v_{7}^{s}+v_{9}^{s}+2v_{10}^{s}+2v_{11}^{s}+v_{16}^{s}$ (1)

$\frac{d}{dt}\left[\text{ATP}_{\text{c}}\right]=-v_{2}^{s}-v_{5}^{s}-2v_{6}^{s}-2v_{7}^{s}-v_{9}^{s}-2v_{10}^{s}-2v_{11}^{s}-v_{15}^{s}$ (2)

$\frac{d}{dt}\left[\text{ADP}_{\text{n}}\right]=2v_{14}^{s}-v_{16}^{s}$ (3)

$\frac{d}{dt}\left[\text{ATP}_{\text{n}}\right]=-2v_{14}^{s}+v_{15}^{s}$ (4)

$\frac{d}{dt}\left[\text{H}^{+}_{\text{c}}\right]=v_{1}^{s}$ (5)

$\frac{d}{dt}\left[\text{H}_{2}\text{O}_{\text{c}}\right]=-v_{1}^{s}$ (6)

$\frac{d}{dt}\left[\text{Hog1}_{\text{c}}\right]=-v_{7}^{s}-v_{11}^{s}$ (7)

$\frac{d}{dt}\left[\text{Hog1pp}_{\text{c}}\right]=v_{8}^{s}+v_{12}^{s}-v_{13}^{s}$ (8)

$\frac{d}{dt}\left[\text{Hog1pp}_{\text{n}}\right]=v_{13}^{s}-v_{14}^{s}$ (9)

$\frac{d}{dt}\left[\left(\text{Hog1pp}\cdot\text{Hot1pp}\right)_{\text{n}}\right]=v_{14}^{s}$ (10)

$\frac{d}{dt}\left[\text{Hot1}_{\text{n}}\right]=-v_{14}^{s}$ (11)

$\frac{d}{dt}\left[\text{osmotic\_stress}_{\text{e}}\right]=-v_{1}^{s}$ (12)

$\frac{d}{dt}\left[\text{p}_{\text{c}}\right]=v_{1}^{s}$ (13)

$\frac{d}{dt}\left[\text{Pbs2}_{\text{c}}\right]=-v_{6}^{s}-v_{10}^{s}$ (14)

$\frac{d}{dt}\left[\text{Sln1}_{\text{c}}\right]=v_{1}^{s}-v_{3}^{s}$ (15)

$\frac{d}{dt}\left[\text{Sln1\_ex}_{\text{c}}\right]=v_{3}^{s}$ (16)

$\frac{d}{dt}\left[\text{Sln1p}_{\text{c}}\right]=-v_{1}^{s}$ (17)

$\frac{d}{dt}\left[\text{Ssk1}_{\text{c}}\right]=-v_{3}^{s}-v_{4}^{s}$ (18)

$\frac{d}{dt}\left[\text{Ssk1\_osmosis}_{\text{c}}\right]=v_{3}^{s}-v_{5}^{s}-v_{9}^{s}$ (19)

$\frac{d}{dt}\left[\text{Ssk1\_osmosis\_ex}_{\text{c}}\right]=v_{5}^{s}+v_{9}^{s}$ (20)

$\frac{d}{dt}\left[\text{Ssk1p}_{\text{c}}\right]=v_{4}^{s}$ (21)

$\frac{d}{dt}\left[\text{Ssk2}_{\text{c}}\right]=-v_{5}^{s}$ (22)

$\frac{d}{dt}\left[\text{Ssk2p}_{\text{c}}\right]=v_{5}^{s}-v_{6}^{s}$ (23)

$\frac{d}{dt}\left[\left(\text{Ssk2p}\cdot\text{Pbs2pp}\right)_{\text{c}}\right]=v_{6}^{s}-v_{7}^{s}$ (24)

$\frac{d}{dt}\left[\left(\text{Ssk2p}\cdot\text{Pbs2pp\_ex}\right)_{\text{c}}\right]=v_{8}^{s}$ (25)

$\frac{d}{dt}\left[\left(\text{Ssk2p}\cdot\text{Pbs2pp}\cdot\text{Hog1pp}\right)_{\text{c}}\right]=v_{7}^{s}-v_{8}^{s}$ (26)

$\frac{d}{dt}\left[\text{Ssk22}_{\text{c}}\right]=-v_{9}^{s}$ (27)

$\frac{d}{dt}\left[\text{Ssk22p}_{\text{c}}\right]=v_{9}^{s}-v_{10}^{s}$ (28)

$\frac{d}{dt}\left[\left(\text{Ssk22p}\cdot\text{Pbs2pp}\right)_{\text{c}}\right]=v_{10}^{s}-v_{11}^{s}$ (29)

$\frac{d}{dt}\left[\left(\text{Ssk22p}\cdot\text{Pbs2pp\_ex}\right)_{\text{c}}\right]=v_{12}^{s}$ (30)

$\frac{d}{dt}\left[\left(\text{Ssk22p}\cdot\text{Pbs2pp}\cdot\text{Hog1pp}\right)_{\text{c}}\right]=v_{11}^{s}-v_{12}^{s}$ (31)

$\frac{d}{dt}\left[\text{Ypd1}_{\text{c}}\right]=-v_{2}^{s}+v_{4}^{s}$ (32)

$\frac{d}{dt}\left[\text{Ypd1p}_{\text{c}}\right]=v_{2}^{s}-v_{4}^{s}$ (33)

**Table S.3.** Kinetic equations of the representative metabolic network.

| **Name** | **Reaction** | **Kinetic Equation** |
| --- | --- | --- |
| $v_{1}^{m}$ | $\left[\text{Glc}_{\text{e}}\right]+\left[\text{H}^{+}_{\text{e}}\right]\to\left[\text{Glc}_{\text{c}}\right]+\left[\text{H}^{+}_{\text{c}}\right]$ | $S_{u}\cdot\left[\text{Glc}_{\text{e}}\right]$ |
| $v_{2}^{m}$ | $\left[\text{Glc}_{\text{c}}\right]+\left[\text{ATP}_{\text{c}}\right]\to$ $\left[\text{Glc}_{\text{c}}\right]+\left[\text{ADP}_{\text{c}}\right]$ | $\frac{k_{2}}{1+\frac{k_{21}}{\left[\text{ATP}_{\text{c}}\right]}\left(1+\frac{\left[\text{ADP}_{\text{c}}\right]}{k_{22}}\right)+\frac{k_{23}}{\left[\text{Glc}_{\text{c}}\right]}+\frac{k_{21}}{\left[\text{ATP}_{\text{c}}\right]}\cdot\frac{k_{23}}{\left[\text{Glc}_{\text{c}}\right]}\left(1+\frac{\left[\text{ADP}_{\text{c}}\right]}{k_{22}}\right)}$ |
| $v_{3}^{m}$ | $\left[\text{G6P}_{\text{c}}\right]+\left[\text{ATP}_{\text{c}}\right]\to$ $\left[\text{FBP}_{\text{c}}\right]+\left[\text{ADP}_{\text{c}}\right]$ | $k_{3}\cdot\frac{\left[\text{ATP}_{\text{c}}\right]}{k_{31}+\left[\text{ATP}_{\text{c}}\right]}\cdot\frac{\left[\text{G6P}_{\text{c}}\right]}{k_{32}+\left[\text{G6P}_{\text{c}}\right]}$ |
| $v_{4}^{m}$ | $\left[\text{FBP}_{\text{c}}\right]\to\left[\text{DHAP}_{\text{c}}\right]+\left[\text{GAP}_{\text{c}}\right]$ | $k_{4}\cdot\frac{\left[\text{FBP}_{\text{c}}\right]-\frac{\left[\text{GAP}_{\text{c}}\right]\left[\text{DHAP}_{\text{c}}\right]}{K_{4}}}{k_{41}+\left[\text{FBP}_{\text{c}}\right]+\frac{k_{42}\cdot\left[\text{GAP}_{\text{c}}\right]}{K_{4}\cdot k_{43}}+\frac{k_{44}\cdot\left[\text{DHAP}_{\text{c}}\right]}{K_{4}\cdot k_{43}}+\frac{\left[\text{FBP}_{\text{c}}\right]\cdot\left[\text{GAP}_{\text{c}}\right]}{K_{41}}+\frac{\left[\text{GAP}_{\text{c}}\right]\cdot\left[\text{DHAP}_{\text{c}}\right]}{K_{4}\cdot k_{43}}}$ |
| $v_{5}^{m}$ | $\left[\text{DHAP}_{\text{c}}\right]+\left[\text{NADH}_{\text{c}}\right]\to$ $\left[\left(\text{Glyc}\cdot\text{p}\right)_{\text{c}}\right]+\left[\text{H}^{+}_{\text{c}}\right]$ | $k_{5}\cdot\frac{\left[\text{DHAP}_{\text{c}}\right]}{k_{51}+\left[\text{DHAP}_{\text{c}}\right]}\cdot\frac{\left[\text{NADH}_{\text{c}}\right]}{k_{52}+\left[\text{NADH}_{\text{c}}\right]}$ |
| $v_{6}^{m}$ | $\left[\left(\text{Glyc}\cdot\text{p}\right)_{\text{c}}\right]+\left[\text{H}_{2}\text{O}_{\text{c}}\right]\to$ $\left[\text{Glyc}_{\text{c}}\right]+\left[\text{p}_{\text{c}}\right]$ | $k_{6}\cdot\left[\left(\text{Glyc}\cdot\text{p}\right)_{\text{c}}\right]$ |
| $v_{7}^{m}$ | $\left[\text{Glyc}_{\text{c}}\right]\to\left[\text{Glyc}_{\text{e}}\right]$ | $k_{7}\cdot\left[\text{Glyc}_{\text{e}}\right]$ |

**Table S.4.** Rate constants of the representative metabolic network.

| **Parameter** | **Value** | **Unit** | **Reference** | **Parameter** | **Value** | **Unit** | **Reference** |
| --- | --- | --- | --- | --- | --- | --- | --- |
| $k_{2}^{m}$ | 1.777 | $[\frac{mM}{s}]$ | [2], Eq. 5.15 | $k_{21}^{m}$ | 0.2 | $[mM]$ | [2], Eq. 5.15 |
| $k_{22}^{m}$ | 1.2 | $[mM]$ | [2], Eq. 5.15 | $k_{23}^{m}$ | 0.2 | $[mM]$ | [2], Eq. 5.15 |
| $k_{3}^{m}$ | 0.895 | $[\frac{mM}{s}]$ | [2], Eq. 5.16 | $k_{31}^{m}$ | 0.01 | $[mM]$ | [2], Eq. 5.16 |
| $k_{32}^{m}$ | 0.012 | $[mM]$ | [2], Eq. 5.16 | $k_{4}^{m}$ | 1867.4 | $[\frac{mM}{s}]$ | [2], Eq. 5.17 |
| $K_{4}^{m}$ | 0.81 | $[mM]$ | [2], Eq. 5.17 | $k_{41}^{m}$ | 10.0 | $[mM]$ | [2], Eq. 5.17 |
| $k_{5}^{m}$ | 1.5 | $[\frac{mM}{s}]$ | [2], Eq. 5.18 | $k_{51}^{m}$ | 1.2 | $[mM]$ | [2], Eq. 5.18 |
| $k_{6}^{m}$ | 1.12 | $[s^{-1}]$ | [2], Eq. 5.28 | $k_{7}^{m}$ | 1.12 | $[s^{-1}]$ | [2], Eq. 5.28 |

**Ordinary differential equations for the representative metabolic network:**

$\frac{d}{dt}\left[\text{Glc}_{\text{e}}\right]=-v_{1}^{m}$ (34)

$\frac{d}{dt}\left[\text{Glc}_{\text{c}}\right]=v_{1}^{m}-v_{2}^{m}$ (35)

$\frac{d}{dt}\left[\text{G6P}_{\text{c}}\right]=v_{2}^{m}-v_{3}^{m}$ (36)

$\frac{d}{dt}\left[\text{FBP}_{\text{c}}\right]=v_{3}^{m}-v_{4}^{m}$ (37)

$\frac{d}{dt}\left[\text{DHAP}_{\text{c}}\right]=v_{4}^{m}-v_{5}^{m}$ (38)

$\frac{d}{dt}\left[\text{GAP}_{\text{c}}\right]=v_{4}^{m}$ (39)

$\frac{d}{dt}\left[\left(\text{Glyc}\cdot\text{p}\right)_{\text{c}}\right]=v_{5}^{m}-v_{6}^{m}$ (40)

$\frac{d}{dt}\left[\text{Glyc}_{\text{c}}\right]=v_{6}^{m}-v_{7}^{m}-v_{\text{reg}}$ (41)

$\frac{d}{dt}\left[\text{Glyc}_{\text{e}}\right]=v_{7}^{m}$ (42)

$\frac{d}{dt}\left[\text{ATP}_{\text{c}}\right]=-v_{2}^{m}-v_{3}^{m}$ (43)

$\frac{d}{dt}\left[\text{ADP}_{\text{c}}\right]=v_{2}^{m}+v_{3}^{m}$ (44)

$\frac{d}{dt}\left[\text{NADH}_{\text{c}}\right]=-v_{5}^{m}$ (45)

$\frac{d}{dt}\left[\text{NAD}^{+}_{\text{c}}\right]=v_{5}^{m}$ (46)

$\frac{d}{dt}\left[\text{p}_{\text{c}}\right]=v_{6}^{m}$ (47)

$\frac{d}{dt}\left[\text{H}_{2}\text{O}_{\text{e}}\right]=-v_{6}^{m}$ (48)

$\frac{d}{dt}\left[\text{H}^{+}_{\text{c}}\right]=v_{1}^{m}+v_{5}^{m}$ (49)

**Ordinary differential equations for the representative regulatory network:**

$v_{\text{reg}}=k_{\text{poly}}\cdot\left[\text{Glyc}_{\text{c}}\right],k_{\text{poly}}=10^{-4}\frac{1}{\mu\text{M}\cdot\text{s}}$ (50)

$v_{\deg\text{,Stl1}}=k_{\deg\text{,Stl1}}\cdot\left[\text{Stl1}\right],k_{\text{deg,Stl1}}=10^{-5}\frac{1}{\text{s}}$ (51)

$v_{\deg\text{,Gpp2}}=k_{\deg\text{,Gpp2}}\cdot\left[\text{Gpp2}\right],k_{\text{deg,Gpp2}}=10^{-5}\frac{1}{\text{s}}$ (52)

$v_{\deg\text{,Gpd1}}=k_{\deg\text{,Gpd1}}\cdot\left[\text{Gpd1}\right],k_{\text{deg,Gpd1}}=10^{-5}\frac{1}{\text{s}}$ (53)

$\frac{d}{dt}\left[\text{Stl1}\right]=v_{\text{reg}}-v_{\deg\text{,Stl1}}$ (54)

$\frac{d}{dt}\left[\text{Gpp2}\right]=v_{\text{reg}}-v_{\deg\text{,Gpp2}}$ (55)

$\frac{d}{dt}\left[\text{Gpd1}\right]=v_{\text{reg}}-v_{\deg\text{,Gpd1}}$ (56)
